# Supplementary material for: Mechanisms of Tumor-Induced Lymphovascular Niche Formation in Draining Lymph Nodes
Source: Cell Rep. 2018 Dec 26;25(13):3554–3563.e4. doi: 10.1016/j.celrep.2018.12.002 (PMC6315107; doi:10.1016/j.celrep.2018.12.002)
Supplement: Document S1. Figures S1–S4 and Tables S1 and S2 [file mmc1.pdf]

Cell Reports, Volume *25*

Supplemental Information

Mechanisms of Tumor-Induced Lymphovascular Niche Formation in Draining Lymph Nodes

Catharina D. Commerford, Lothar C. Dieterich, Yuliang He, Tanja Hell, Javier A. Montoya-Zegarra, Simon F. Noerrelykke, Erica Russo, Martin Röcken, and Michael Detmar

Figure S1:

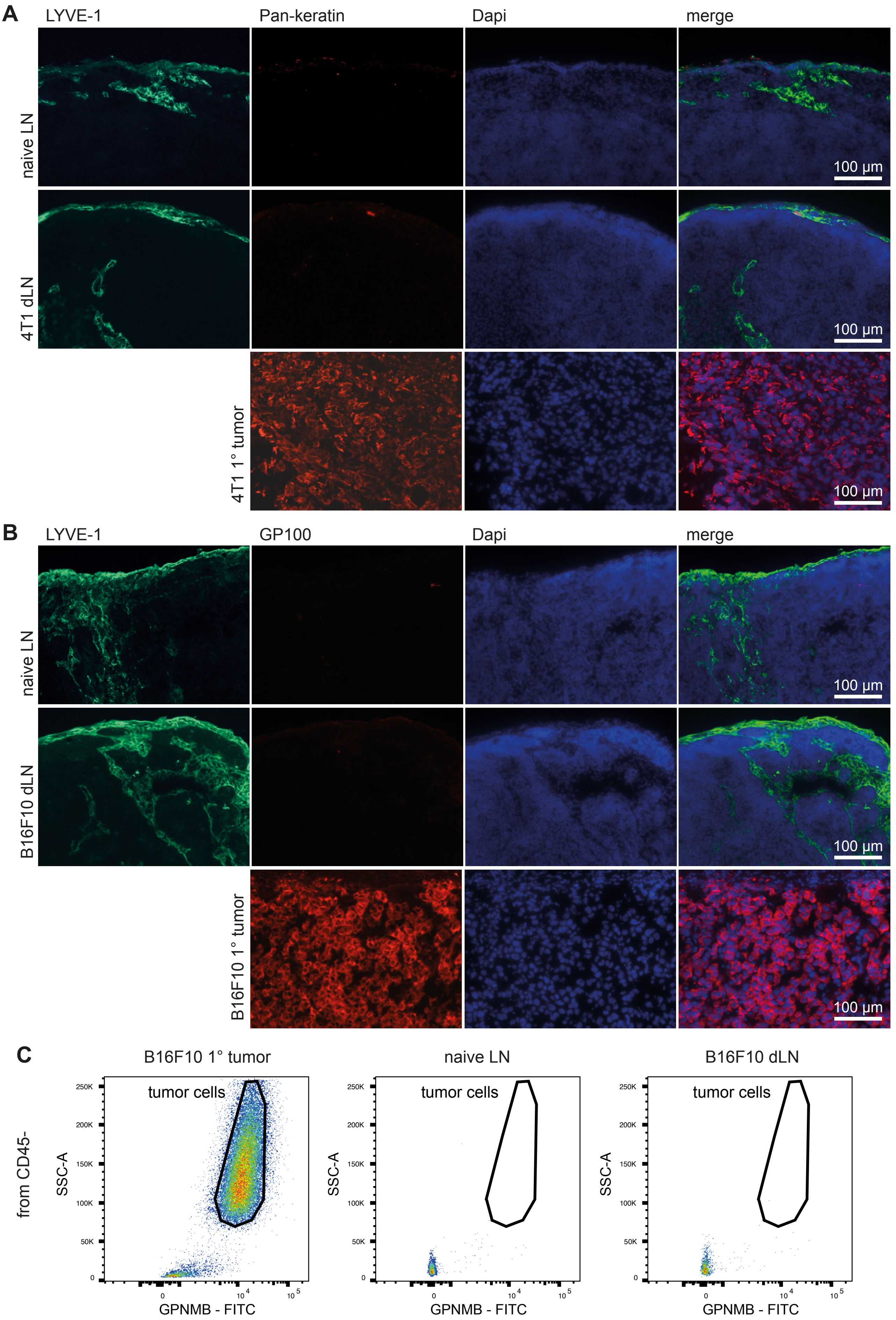
A
LYVE-1
Pan-keratin
Dapi
merge
naive LN
4T1 dLN
4T1 1° tumor
100 µm
B
LYVE-1
GP100
Dapi
merge
naive LN
B16F10 dLN
B16F10 1° tumor
100 µm
C
B16F10 1° tumor
naive LN
B16F10 dLN
from CD45-
SSC-A
tumor cells
GPNMB - FITC

Figure S1. No metastatic tumor cells in 4T1 and B16F10 tumor-draining LNs. Related to Figure 1.
(A and B) Representative immunofluorescence images of naïve LNs, tumor-draining LNs, and primary tumors stained for (A) pan-keratin or (B) the melanoma marker gp100. The pan-keratin staining shows some artefacts in both naïve, as well as 4T1 tumor-draining LNs. (C) Representative FACS plots showing GPNMB+ melanoma cells in B16F10 primary tumors but not in naïve or tumor-draining LNs. Cells were pre-gated on single, living, CD45- cells.

Figure S2:

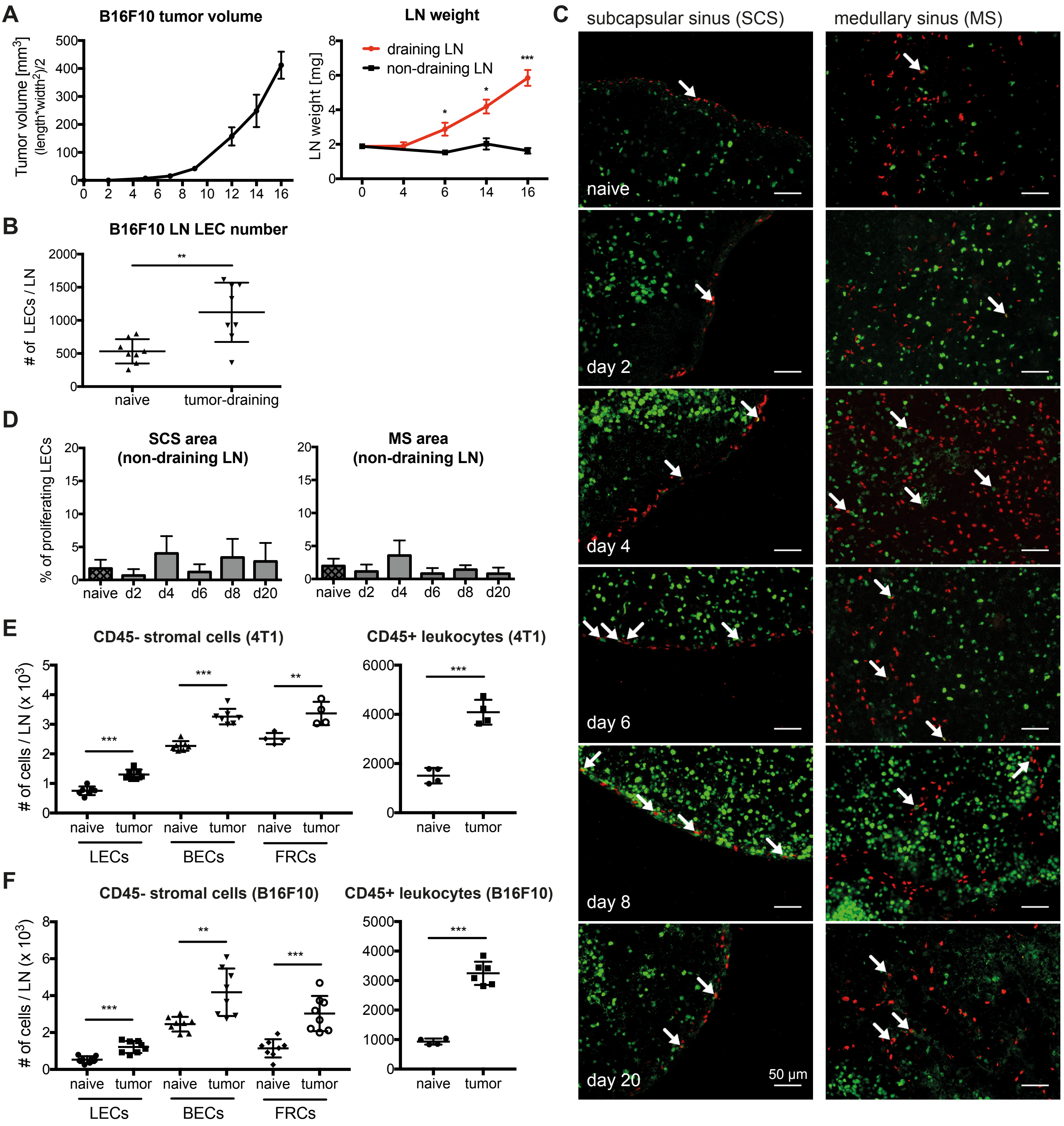
A
B16F10 tumor volume
Tumor volume [mm3] (length*width2)/2
LN weight
draining LN
non-draining LN
LN weight [mg]
B
B16F10 LN LEC number
# of LECs / LN
naive
tumor-draining
C
subcapsular sinus (SCS)
medullary sinus (MS)
naive
day 2
day 4
day 6
day 8
day 20
50 µm
D
SCS area (non-draining LN)
MS area (non-draining LN)
% of proliferating LECs
naive d2 d4 d6 d8 d20
E
CD45- stromal cells (4T1)
CD45+ leukocytes (4T1)
# of cells / LN (x 103)
naive tumor naive tumor naive tumor
LECs
BECs
FRCs
F
CD45- stromal cells (B16F10)
CD45+ leukocytes (B16F10)

Figure S2. LN weight and LEC proliferation increase over time in tumor-draining LNs. Related to Figure 1.

(A) B16F10 primary tumor growth assessed by caliper measurements *in vivo* and *ex vivo* inguinal LN weight.

(B) FACS quantification of LEC numbers in naïve and B16F10 tumor-draining LNs at day 14.

(C) Representative images of the subcapsular and medullary sinus (SCS & MS) of 4T1 tumor-draining LNs at different time points stained for Prox1 (red) and Ki67 (green), with arrows indicating proliferating LECs.

(D) Quantification of proliferation in the SCS & MS of non-draining contralateral inguinal LNs in 4T1 mice.

(E and F) FACS quantification shows increased LEC, BEC, FRC and leukocyte numbers in (E) 4T1 and (F) B16F10 tumor-draining LNs compared to the respective naïve control LNs. Statistical significance was determined with the unpaired student t-test (A, B, E, F) or one-way ANOVA (D).

Figure S3:

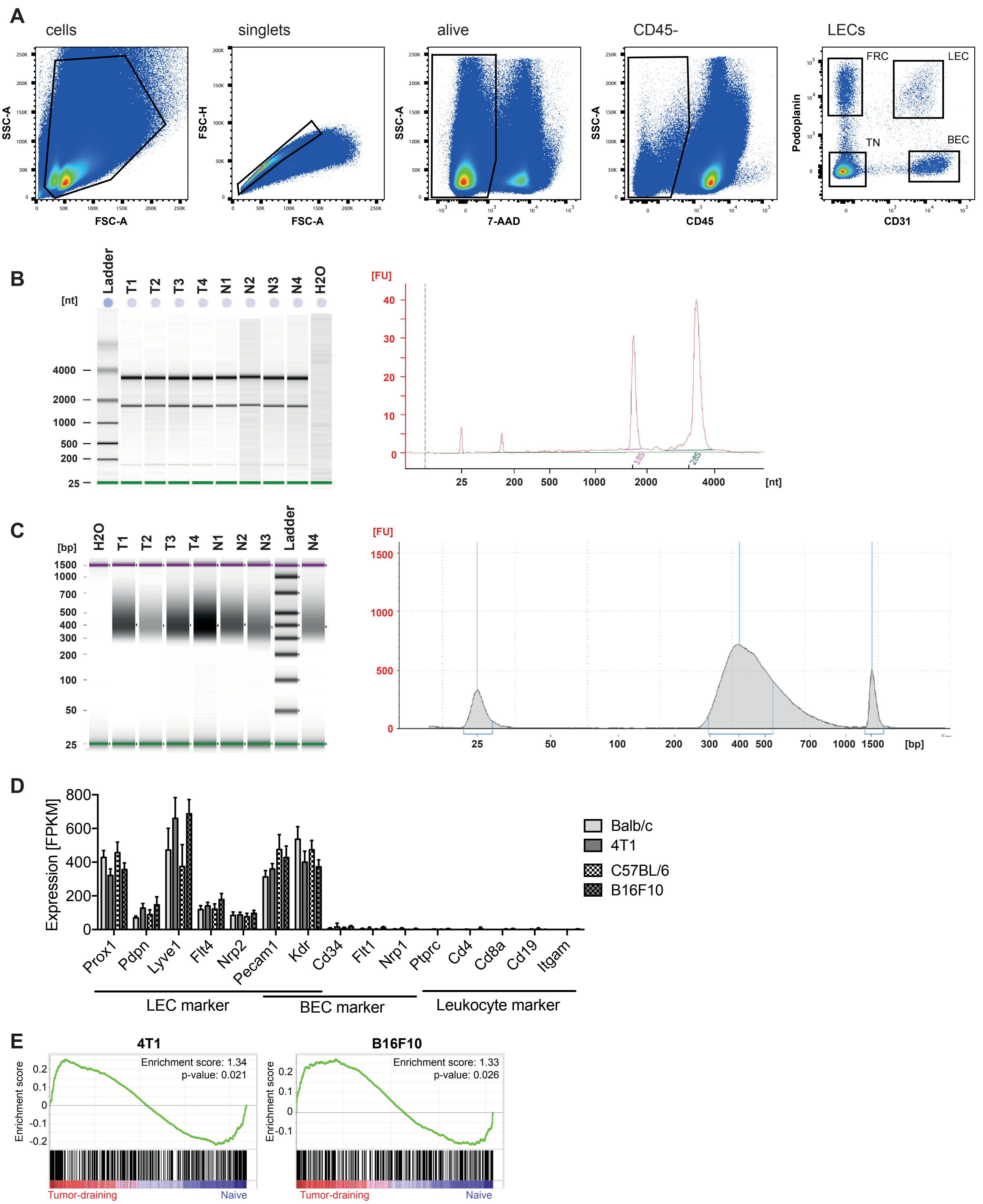
A
cells
singlets
alive
CD45-
LECs
SSC-A
FSC-A
FSC-H
7-AAD
CD45
Podoplanin
CD31
FRC
LEC
TN
BEC
B
Ladder
T1
T2
T3
T4
N1
N2
N3
N4
H2O
[nt]
4000
2000
1000
500
200
25
[FU]
40
30
20
10
0
18S
28S
[nt]
C
H2O
T1
T2
T3
T4
N1
N2
N3
Ladder
N4
[bp]
1500
1000
700
500
400
300
200
100
50
25
[FU]
[bp]
D
Expression [FPKM]
800
600
400
200
0
Balb/c
4T1
C57BL/6
B16F10
Prox1
Pdpn
Lyve1
Flt4
Nrp2
Pecam1
Kdr
Cd34
Flt1
Nrp1
Ptprc
Cd4
Cd8a
Cd19
Itgam
LEC marker
BEC marker
Leukocyte marker
E
4T1
B16F10
Enrichment score
Enrichment score: 1.34
p-value: 0.021
Enrichment score: 1.33
p-value: 0.026
Tumor-draining
Naive

Figure S3. Quality controls for RNA sequencing and gene set enrichment analysis. Related to Figure 2. (A) FACS gating strategy for LN stromal cells. (B and C) Representative gels and histograms of (B) RNA and (C) cDNA samples from naïve and 4T1 tumor mice. (B) RNA quality as assessed by Agilent Bioanalyzer (RIN 7.8-10). (C) cDNA libraries with fragments between 300-500 bp length and without contaminations as tested on an Agilent TapeStation. (D) Relative expression of LEC-, BEC- and leukocyte-specific genes as measure for the high purity of sorted and sequenced LN LECs. (E) Gene set enrichment analysis of previously published sprouting tip cell-associated genes (del Toro et al., 2010; Strasser et al., 2010) among the differentially expressed genes in LN LECs in the 4T1 (left panel) and B16F10 (right panel) tumor models.

Figure S4:

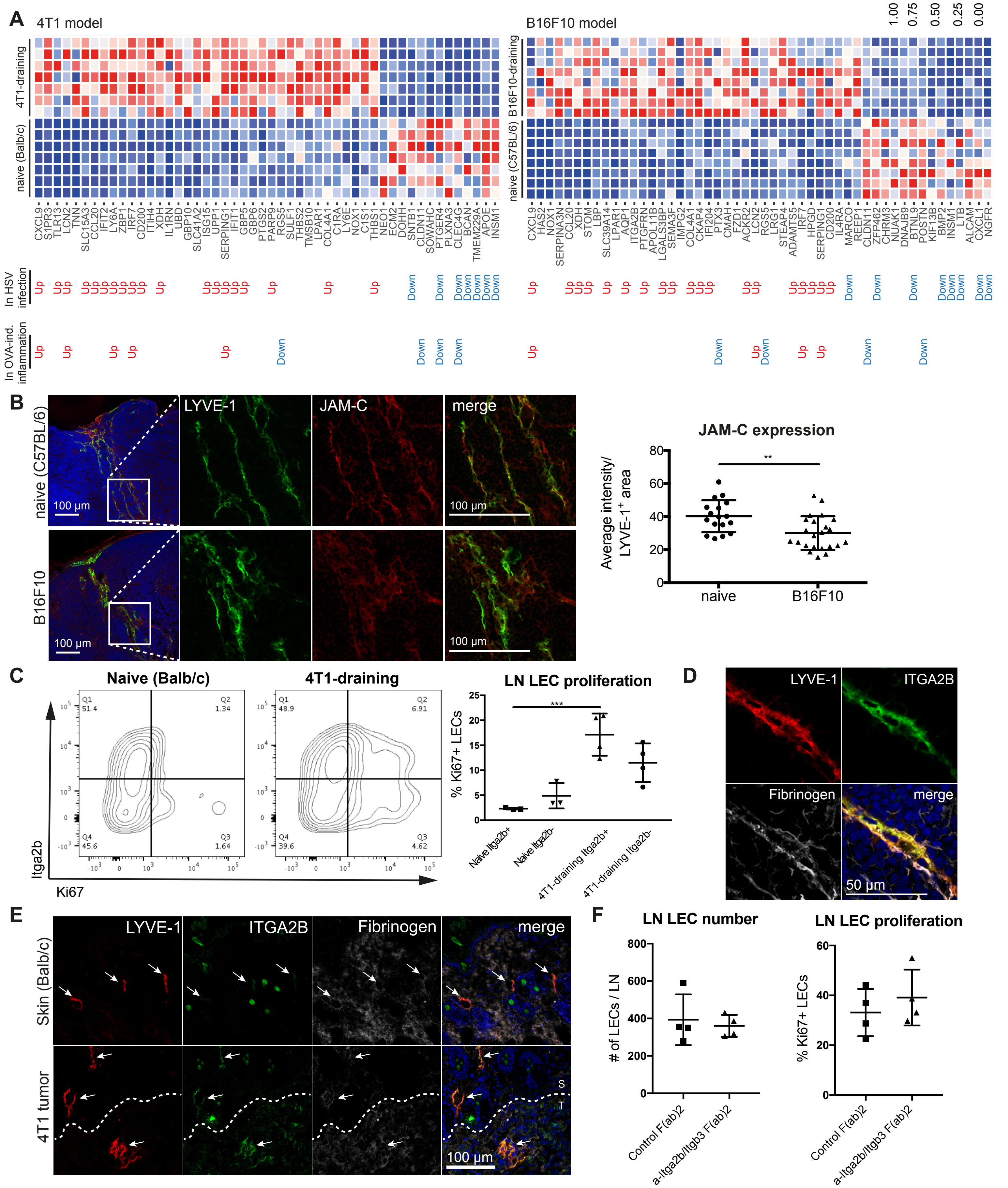
A
4T1 model
B16F10 model
value
1.00
0.75
0.50
0.25
0.00
4T1-draining
naive (Balb/c)
B16F10-draining
naive (C57BL/6)
In HSV infection
In OVA-ind. inflammation
Up
Down
B
LYVE-1
JAM-C
merge
100 µm
JAM-C expression
Average intensity/ LYVE-1+ area
naive
B16F10
C
Naive (Balb/c)
4T1-draining
Q1 51.4
Q2 1.34
Q3 1.64
Q4 45.6
Q1 48.9
Q2 6.91
Q3 4.62
Q4 39.6
Itga2b
Ki67
LN LEC proliferation
% Ki67+ LECs
Naive Itga2b+
Naive Itga2b-
4T1-draining Itga2b+
4T1-draining Itga2b-
D
LYVE-1
ITGA2B
Fibrinogen
merge
50 µm
E
Skin (Balb/c)
4T1 tumor
S
T
F
LN LEC number
# of LECs / LN
Control F(ab)2
a-Itga2b/Itgb3 F(ab)2
% Ki67+ LECs

Figure S4. Differential gene expression in LECs from tumor-draining LNs compared to naïve controls. Related to Figures 3 and 4.

(A) Heat map of top 50 most differentially expressed genes is shown for both tumor models separately (left: 4T1 vs. naïve Balb/c; right: B16F10 vs. naïve C57BL/6). Significant up- or downregulation in previously published gene expression datasets of LN LECs after HSV infection (Gregory et al., 2017) or after ovalbumin injection in OT-1 transferred mice (Malhotra et al., 2012) is indicated. (B) Representative immunofluorescence images and quantification of JAM-C staining intensity within LYVE-1 positive lymphatic structures of naïve and B16F10 tumor-draining LNs. Each dot represents one image, n = 8 individual LNs per group. Statistical significance was determined by the unpaired Student's t-test. (C) FACS analysis of LN LECs in naïve Balb/c and 4T1-bearing mice (day 10) for expression of integrin αIIb and Ki67. Example plots (left panels) and quantification (right panels, n = 3-4 mice / group) are shown. (D) Representative immunofluorescence image of fibrinogen localization around an integrin αIIb expressing lymphatic vessel in a 4T1 draining LN (day 20). (E) Representative immunofluorescence images of integrin αIIb expression and fibrinogen deposition in lymphatic vessels in control skin and primary 4T1 tumors (day 20). (F) FACS analysis of draining LN LEC numbers and proliferation rate (as determined by Ki67 staining) in 4T1-bearing mice (day 10) treated daily from day 4 on with a rat IgG2b F(ab)2 fragment or an integrin αIIb blocking F(ab)2 fragment.

Table S1:

		FACS sorted cell number				RNA extraction			DNA library prep	
	**sample**	**LEC**	**BEC**	**FRC**	**TN**	**RIN**	**RNA conc. [pg/µl]**	**total RNA [ng]**	**DNA conc. [pg/µl]**	**Molarity [nM]**
	**N1**	5016	8431	/	/	8.6	251	2.51	6350	24.40
	**N2**	2076	10135	/	/	9.9	234	2.34	5480	23.00
	**N3**	3105	8532	/	/	8.6	459	4.59	2630	10.60
**Balb/c**	**N4**	4225	10299	/	/	7.8	221	2.21	5130	20.00
	**N5**	2768	11378	10034	68533	8.7	499	4.99	820	3.15
	**N6**	3049	8810	12110	57358	9	660	6.60	2970	11.60
	**N7**	2788	8985	9794	66826	8.4	548	5.48	4450	17.20
	**average**	3290	9510	10646	64239					
	**T1**	1832	7569	/	/	9.8	382	3.82	806	3.31
	**T2**	2399	6682	/	/	9.3	462	4.62	1690	6.92
	**T3**	2201	6358	/	/	10	401	4.01	3320	13.40
**4T1**	**T4**	2487	5443	/	/	7.9	709	7.09	1960	7.50
	**T5**	1313	5536	5063	89048	8.8	236	2.36	4280	16.50
	**T6**	2293	5987	6139	91335	8	535	5.35	3310	13.40
	**T7**	2719	6064	7999	94794	9.1	384	3.84	4170	15.00
	**average**	2178	6234	6400	91726					
	**N1**	1415	7762	5866	11482	8.4	238	2.38	310	1.24
	**N2**	3193	10056	8735	65806	9.3	1033	10.33	2080	7.98
	**N3**	1925	10391	3299	21926	9.0	481	4.81	2660	10.70
**C57BL/6**	**N4**	1024	1856	929	14359	7.9	199	1.99	2080	8.71
	**N5**	2134	13047	3716	68532	8.4	357	3.57	3710	15.20
	**N6**	1967	12181	5175	50218	8.3	667	6.67	1080	4.42
	**N7**	2389	11294	4640	62914	8.5	727	7.27	5390	22.40
	**N8**	3006	7600	5142	42456	8.6	709	7.09	9	0.04
	**average**	2132	9273	4688	42212					
	**T1**	1524	5490	5363	19391	9.5	369	3.69	2350	9.48
	**T2**	719	3995	2991	15776	8.6	280	2.80	1900	7.52
	**T3**	2653	5131	11385	95043	9.5	1035	10.35	2200	9.14
**B16F10**	**T4**	3071	7339	6376	103462	9.5	1015	10.15	2920	11.60
	**T5**	1849	8883	4414	64414	8.9	592	5.92	3410	14.20
	**T6**	3228	12168	7462	32195	9.6	816	8.16	8900	35.10
	**T7**	1860	5835	3227	61444	8.9	547	5.47	3670	13.90
	**T8**	3045	11057	7245	117991	8.7	688	6.88	6370	26.00
	**average**	2244	7487	6058	63715					

Table S1. List of all samples used for RNA sequencing. Related to Figure 2.
Number of sorted LN stromal cells, LEC RNA quality (RIN) and quantity, and cDNA concentration in the libraries are shown for all samples used in this study. n = 7-8 samples per group.

Table S2:

Cluster 1

BP (Biological Process)

Term	ID	p	N
immune system process	GO:0002376	7.252306e-09	18/187
positive regulation of macrophage activation	GO:0043032	3.531137e-06	4/7
innate immune response	GO:0045087	3.699133e-07	15/168
cellular response to interferon-gamma	GO:0071346	1.036803e-06	6/20
cell adhesion	GO:0007155	5.197905e-07	24/247
defense response	GO:0006952	9.695147e-06	5/17
adhesion of symbiont to host	GO:0044406	1.235389e-05	4/9
negative regulation of endothelial cell proliferation	GO:0001937	3.005367e-05	5/21
negative regulation of angiogenesis	GO:0016525	3.461842e-05	6/35

Molecular Function (MF)

Term	ID	p	N
extracellular matrix binding	GO:0050840	5.97E-06	5/16
scavenger receptor activity	GO:0005044	1.52E-05	5/19
calcium ion binding	GO:0005509	2.55E-05	15/244
chemorepellent activity	GO:0045499	5.23E-05	3/5
collagen binding	GO:0005518	5.51E-05	6/39
extracellular matrix structural constituent	GO:0005201	8.10E-05	4/14

Cellular Composition (CC)

Term	ID	p	N
extracellular region	GO:0005576	1.39104E-11	33/471
cell surface	GO:0009986	6.5854E-11	25/289
membrane	GO:0016020	5.0668E-09	109/3895
plasma membrane	GO:0005886	2.13435E-07	58/1664
extracellular matrix	GO:0031012	1.13981E-05	10/99
proteinaceous extracellular matrix	GO:0005578	1.81583E-05	11/127
endoplasmic reticulum	GO:0005783	5.45915E-05	32/853

Cluster 2

BP (Biological Process)

Term	ID	p	N
response to cAMP	GO:0051591	6.61424E-06	5/14
SMAD protein signal transduction	GO:0060395	3.74705E-05	6/31
positive regulation of cell differentiation	GO:0045597	4.62223E-05	5/20
transcription, DNA-templated	GO:0006351	6.83504E-05	46/1242
transcription from RNA polymerase II promoter	GO:0006366	1.7366E-05	17/248
negative regulation of transcription	GO:0000122	1.18991E-08	31/471
positive regulation of transcription	GO:0045944	2.49249E-08	35/598
regulation of transcription, DNA templated	GO:0006355	5.73019E-06	51/1308
response to mechanical stimulus	GO:0009612	8.13038E-05	4/12

Molecular Function (MF)

Term	ID	p	N
transcription factor activity, sequence-specific DNA binding	GO:0003700	5.08779E-07	27/436
transcriptional activator activity	GO:0001077	7.30895E-05	11/126
protein binding	GO:0005515	2.70703E-05	88/2845
DNA binding	GO:0003677	5.72488E-05	41/1036
sequence-specific DNA binding	GO:0043565	1.19057E-06	20/271

Cellular Composition (CC)

Term	ID	p	N
nuclear chromatin	GO:0000790	6.73611E-05	11/127

Cluster 3

BP (Biological Process)

Term	ID	p	N
response to virus	GO:0009615	4.43297E-06	5/43
defense response to virus	GO:0051607	7.22143E-10	9/81
cellular response to interferon-beta	GO:0035458	5.61888E-06	4/21
G-protein coupled receptor signaling pathway	GO:0007186	1.00859E-05	7/132
immune system process	GO:0002376	1.0928E-05	8/187
inflammatory response	GO:0006954	5.49557E-05	6/117

Molecular Function (MF)

Term	ID	p	N
chemokine activity	GO:0008009	5.44971E-05	3/13

Cellular Composition (CC)

Term	ID	p	N
extracellular region	GO:0005576	2.33269E-06	13/471

Cluster 4

BP (Biological Process): N/A

Molecular Function (MF): N/A

Cellular Composition (CC): N/A

Cluster 5

BP (Biological Process): N/A

Molecular Function (MF): N/A

Cellular Composition (CC)

Term	ID	p	N
extracellular space	GO:0005615	1.93289E-05	6/462

Cluster 6

BP (Biological Process)

Term	ID	p	N
cell division	GO:0051301	1.20509E-05	5/259
mitotic nuclear division	GO:0007067	8.49154E-05	4/194

Molecular Function (MF): N/A

Cellular Composition (CC): N/A

Table S2. Complete list of gene ontologies (GOs) associated with the clusters in Figure 3A. Related to Figure 3.

Movie S1. Optical sectioning reveals the lymphatic network architecture in resting LNs of naïve mice. Related to Figure 1.
Consecutive 3D light-sheet microscope images of a whole naïve LN stained for the lymphatic marker LYVE-1.

Movie S2. Optical sectioning reveals altered lymphatic network architecture in 4T1 tumor-draining LNs. Related to Figure 1.
Consecutive 3D light-sheet microscope images of a whole 4T1 tumor-draining LN stained for the lymphatic marker LYVE-1.
