# Supplementary material for: Mechanisms of Tumor-Induced Lymphovascular Niche Formation in Draining Lymph Nodes
Source: Cell Rep. 2018 Dec 26;25(13):3554–3563.e4. doi: 10.1016/j.celrep.2018.12.002 (PMC6315107; doi:10.1016/j.celrep.2018.12.002)
Supplement: Document S2. Article plus Supplemental Information [file mmc4.pdf]

Cell Reports

Report

Mechanisms of Tumor-Induced Lymphovascular Niche Formation in Draining Lymph Nodes

Graphical Abstract

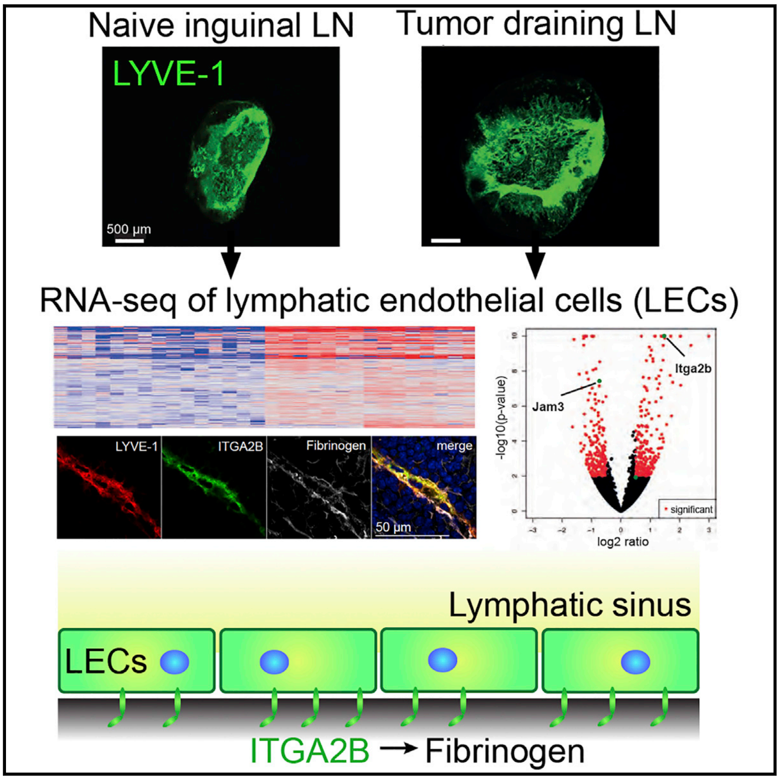

Authors

Catharina D. Commerford,
Lothar C. Dieterich, Yuliang He, ...,
Erica Russo, Martin Röcken,
Michael Detmar

Correspondence

michael.detmar@pharma.ethz.ch

In Brief

Commerford et al. characterize the morphological and molecular changes that occur in lymphatic endothelial cells (LECs) in tumor-draining lymph nodes (LNs) in two distinct tumor models. They show an upregulation of integrin αIIb in LN LECs and find that integrin αIIb mediates LEC adhesion to fibrinogen.

Highlights

- Lymph node lymphatic endothelial cells (LN LECs) dramatically react to tumor stimuli
- Cell adhesion molecules are among the strongest differentially regulated genes
- Itga2b is upregulated and associated with fibrinogen in tumor-draining LN LECs
- Itga2b mediates adhesion of LN LECs to fibrinogen

Commerford et al., 2018, Cell Reports *25*, 3554–3563
December 26, 2018 © 2018 The Author(s).
https://doi.org/10.1016/j.celrep.2018.12.002

CellPress

OPEN ACCESS
CellPress

Cell Reports
Report

Mechanisms of Tumor-Induced Lymphovascular Niche Formation in Draining Lymph Nodes

Catharina D. Commerford,[1,4] Lothar C. Dieterich,[1,4] Yuliang He,[1] Tanja Hell,[1] Javier A. Montoya-Zegarra,[1,2] Simon F. Noerrelykke,[2] Erica Russo,[1] Martin Röcken,[3] and Michael Detmar[1,5,*]
[1]Institute of Pharmaceutical Sciences, ETH Zurich, 8093 Zurich, Switzerland
[2]Scientific Center for Optical and Electron Microscopy, ETH Zurich, 8093 Zurich, Switzerland
[3]Department of Dermatology, Eberhard Karls University, 72076 Tübingen, Germany
[4]These authors contributed equally
[5]Lead Contact
*Correspondence: michael.detmar@pharma.ethz.ch
https://doi.org/10.1016/j.celrep.2018.12.002

SUMMARY

Enlargement of the lymphatic vascular network in tumor-draining lymph nodes (LNs) often precedes LN metastasis, likely providing a lymphovascular niche for tumor cells. We investigated morphological and molecular changes associated with the lymphatic remodeling process, using the 4T1 breast cancer and B16F10 melanoma models. Lymphatic expansion in tumor-draining LNs is mediated by sprouting and proliferation of lymphatic endothelial cells (LECs) as early as 4 days after tumor implantation. RNA sequencing revealed an altered transcriptional profile of LECs from tumor-draining compared to naive LNs with similar changes in both tumor models. Integrin αIIb is upregulated in LECs of tumor-draining LNs and mediates LEC adhesion to fibrinogen *in vitro*. LEC-associated fibrinogen was also detected in LNs *in vivo*, suggesting a role of integrin αIIb in lymphatic remodeling. Together, our results identify specific responses of LN LECs to tumor stimuli and provide insights into the mechanisms of lymphovascular niche formation in tumor-draining LNs.

INTRODUCTION

In many human cancers, including melanoma and breast cancer, tumor-associated lymphangiogenesis predicts increased metastasis and poor clinical outcome (Dieterich and Detmar, 2016; Stacker et al., 2014), and overexpression of (lymph-) angiogenic factors promotes tumor lymphangiogenesis and lymph node (LN) metastasis in experimental models (Mandriota et al., 2001; Skobe et al., 2001; Stacker et al., 2001), which has recently been shown to contribute to systemic metastasis (Brown et al., 2018; Pereira et al., 2018). Lymphangiogenesis also occurs in tumor-draining LNs. For instance, mice overexpressing vascular endothelial growth factor (VEGF)-A or VEGF-C in a model of chemically induced skin carcinogenesis showed prominently enhanced LN lymphangiogenesis, concomitant with a significant increase of LN and distant organ metastasis (Hirakawa et al., 2005, 2007). Importantly, these and other studies using experimental models of nasopharyngeal carcinoma (Qian et al., 2006) and malignant melanoma (García-Caballero et al., 2017; Harrell et al., 2007) have identified the onset of LN lymphatic remodeling prior to the colonization of the LN by metastatic cells. These data suggest that, even in the absence of metastatic tumor cells, tumor-associated lymphangiogenic factors can be drained from the primary tumor or released by locally activated cells to initiate changes in preparation of a pre-metastatic "lymphovascular niche" (Hirakawa et al., 2005; Ogawa et al., 2014; Olmeda et al., 2017). LN lymphangiogenesis has also been found in patients with malignant melanoma and human breast cancer, where the extent of lymphangiogenesis in sentinel LNs predicted an increase in the occurrence of distant LN metastases (Dadras et al., 2005; Pastushenko et al., 2016; Van den Eynden et al., 2007).

Interestingly, accumulating evidence suggests that tumor cells can express receptors for lymphatic endothelial cell (LEC)-produced chemokines that support their migration toward lymphatic vessels and LNs, hijacking physiologic pathways for leukocyte homing (Müller et al., 2001; Wiley et al., 2001). Moreover, LN LECs have been proposed to provide a cancer stem cell niche by producing chemokines that support the survival of cancer cells with stem-like properties and high metastatic potential (Kim et al., 2010; Li et al., 2015). Recent findings indicate that LN LECs can directly modulate immune responses and that the lymphatic system might decisively shape the immune response to the tumor (reviewed in Rouhani et al., 2014). Together, these studies indicate that LECs in the tumor and in draining LNs might play an important role in tumor progression.

Although LN lymphatic remodeling has been described in murine tumor models and human cancers, an extensive morphological and molecular characterization of this process has been lacking. In this study, we thoroughly characterized the expansion of the lymphatic network in tumor-draining LNs over time. We also performed transcriptional profiling of LN LECs isolated from two different murine tumor models and identified a transcriptional profile that is common in both models. Together, these studies provide a comprehensive portrait of the structural

 © 2018 The Author(s).
This is an open access article under the CC BY license (http://creativecommons.org/licenses/by/4.0/).

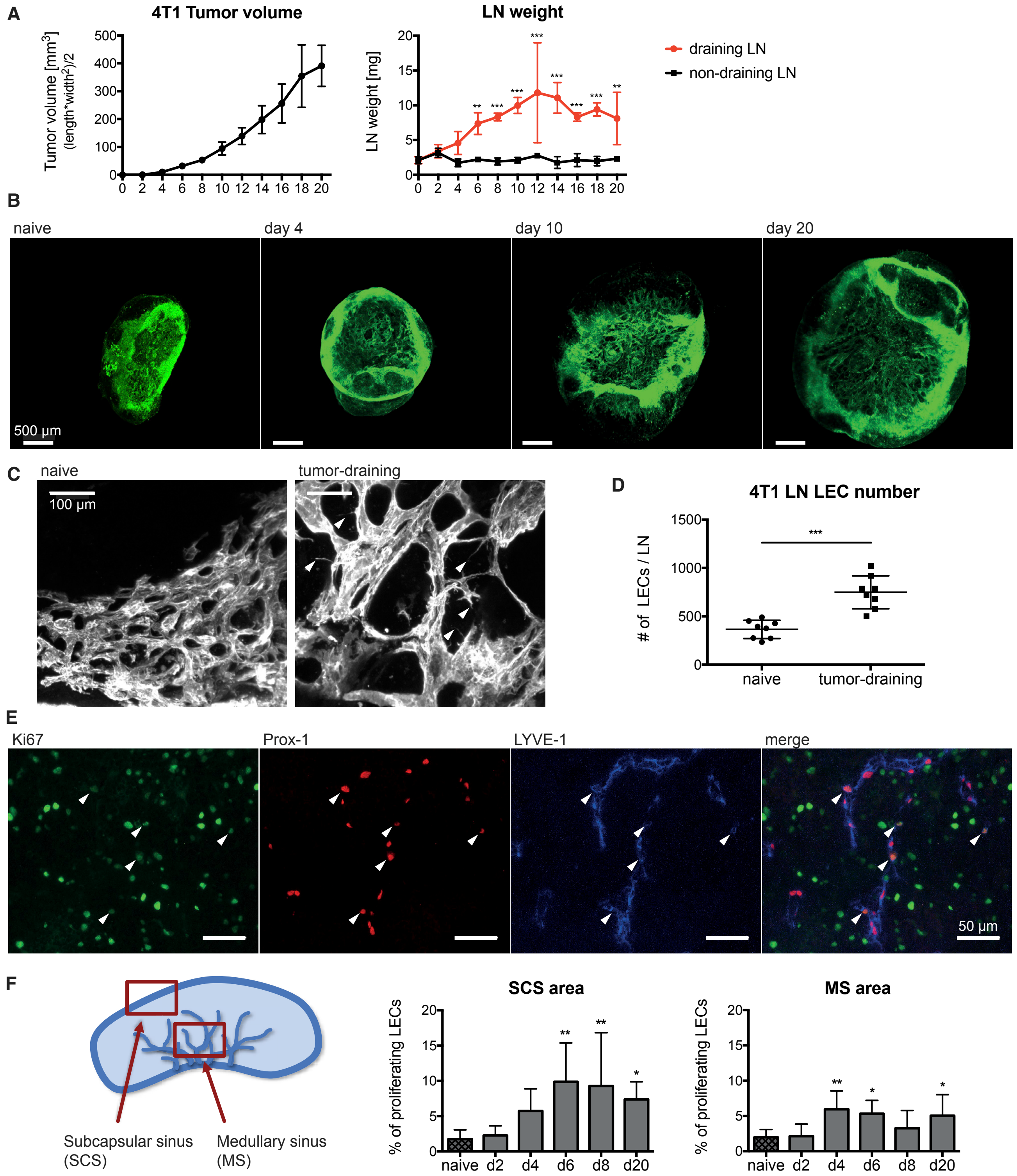

Figure 1. Lymphatic Network Expansion in Tumor-Draining LNs Is Mediated by LEC Sprouting and Proliferation

(A) 4T1 primary tumor growth and *ex vivo* inguinal LN weight.

(B and C) Maximum intensity projections of 3D light-sheet-microscope images of whole LNs stained for the lymphatic marker LYVE-1 (B) at different times or (C) 10 days after 4T1 injection.

(D) FACS quantification of LECs in naive and 4T1 tumor-draining LNs at day 10.

(legend continued on next page)

and molecular adaptations of LN LECs in response to tumor stimuli, and they identify pathways that may regulate these processes.

RESULTS

Lymphatic Network Expansion in Tumor-Draining LNs Is Mediated by LEC Sprouting and Proliferation

We used the murine 4T1 breast cancer and B16F10 melanoma models to investigate morphological and molecular changes of the lymphatic network in tumor-draining LNs over time before the arrival of metastatic tumor cells. No LN metastasis was detected in either model until the last studied time points (Figure S1). Tumor growth in both models was accompanied by an increased weight of the tumor-draining, but not the contralateral non-draining, inguinal LN, indicating a local tumor-mediated effect (Figures 1A and S2A). In the 4T1 model, LN enlargement occurred already around day 4 after tumor cell injection, when tumors were barely palpable, and reached a plateau around day 10 (Figure 1A). In contrast, B16F10 tumor-draining LNs increased in weight more slowly and continued swelling until day 16, when mice had to be sacrificed (Figure S2A).

3D light-sheet microscopy of whole LNs was used to assess 4T1 tumor-induced remodeling of the LN lymphatic network. A massive expansion of the lymphatic network started together with the enlargement of the LN around day 4 and progressed until day 20 (Figure 1B; Videos S1 and S2). Higher magnification images revealed frequent filopodia formation by lymphatic vessels in tumor-draining LNs, which was not observed in naive LNs (Figure 1C). There were also clear alterations of the network architecture, with dilated lymphatic vessels and an expanded network as compared to naive LNs.

Fluorescence-activated cell sorting (FACS) analysis revealed an increased number of LECs in tumor-draining LNs in both tumor models, indicating that the enlargement of lymphatic vessels was at least partially mediated by LEC hyperplasia (Figures 1D and S2B). Stainings of LN sections for LYVE-1, Prox1, and the proliferation marker Ki67 confirmed that the LEC proliferation rate was increased both in the subcapsular sinus and the medullary sinus of 4T1 tumor-draining, but not non-draining, LNs (Figures 1E, 1F, S2C, and S2D). Compared to a baseline LEC proliferation rate of less than 2% in resting LNs of naive mice, proliferation increased at day 4 after tumor cell injection and reached up to 10% in the subcapsular sinus and 6% in the medullary sinus around days 4–6. In accordance with previously published reports (Riedel et al., 2016), we observed that, besides LECs, other stromal cell populations, like blood vascular endothelial cells (BECs) and fibroblastic reticular cells (FRCs), as well as leukocytes expand significantly in tumor-draining LNs (Figures S2E and S2F).

In conclusion, these findings show that the enlargement of tumor-draining LNs is accompanied by a remarkable expansion of stromal cell populations and that the remodeling of the lymphatic network is mediated by lymphatic vessel sprouting and LEC proliferation.

RNA Sequencing Reveals a Distinct Expression Profile of LECs from Tumor-Draining LNs

Given the major structural alterations of lymphatic vessels, we next sought to elucidate how LECs adapt to the environmental changes in tumor-draining LNs on a molecular level. We sorted LECs from 4T1 and B16F10 tumor-draining LNs and their respective naive controls and subjected them to RNA sequencing (Figures 2A, 2B, and S3A–S3C; Table S1). Robust expression of LEC-specific marker genes, but not of blood vessel- or leukocyte-specific markers, confirmed the high purity of the samples that were used for sequencing (Figure S3D). Principal-component analysis (PCA) showed that the samples clustered according to the four experimental groups (Figure 2C). Interestingly, PCA based on all genes resulted in a closer clustering according to the genetic background of the mice (BALB/c versus C57BL/6), whereas PCA based on differentially expressed genes suggested greater similarity by treatment (naive PBS versus tumor). Importantly, about one third of all differentially expressed genes were shared between both models (Figure 2D). These results indicate that LN LECs in different mouse strains have a distinct gene expression profile yet modify this expression in a similar fashion when subjected to tumor-derived stimuli, even if those are derived from very different tumor types (breast cancer and melanoma).

In line with the morphological changes observed in the lymphatic sinuses of tumor-draining LNs (Figure 1C), gene set enrichment analysis showed that the differentially expressed genes in both tumor models were enriched for genes previously associated with sprouting tip cells (del Toro et al., 2010; Strasser et al., 2010; Figure S3E).

Clustering of universally deregulated genes in both models into functionally related groups based on gene ontology highlighted an upregulation of genes involved in cell division, immune-modulatory pathways, and cell adhesion, whereas many genes involved in transcription regulation and differentiation were downregulated (Figures 3A and S4A; Table S2). Interestingly, we observed a considerable overlap with previously published gene expression datasets of LN LECs 6 days after viral infection (Gregory et al., 2017) and some overlap with gene expression datasets of LN LECs in ovalbumin-induced inflammation (Malhotra et al., 2012; Figure S4A), whereas no overlap with LEC migration-associated genes (Williams et al., 2017) was found. For further studies, we focused on genes up- or downregulated in both tumor models that are involved in cell-cell interactions. Especially adhesion molecules play an important

(E and F) Immunofluorescence staining of 4T1 tumor-draining LNs for the lymphatic markers Prox1 and LYVE-1 and the proliferation marker Ki67.
(E) Representative image of a tumor-draining LN with arrowheads indicating proliferating LECs.
(F) Schematic of the analyzed LN areas (left) and quantification of LEC proliferation in these areas (right).
Statistical significance was determined by (A) two-way ANOVA, (D) unpaired Student's t test, or (F) one-way ANOVA. Data are shown as mean with SD and differences were considered statistically significant when $p < 0.05$, as indicated by *$p < 0.05$, **$p < 0.01$, and ***$p < 0.001$. See also Videos S1 and S2 and Figures S1 and S2.

OPEN ACCESS
CellPress

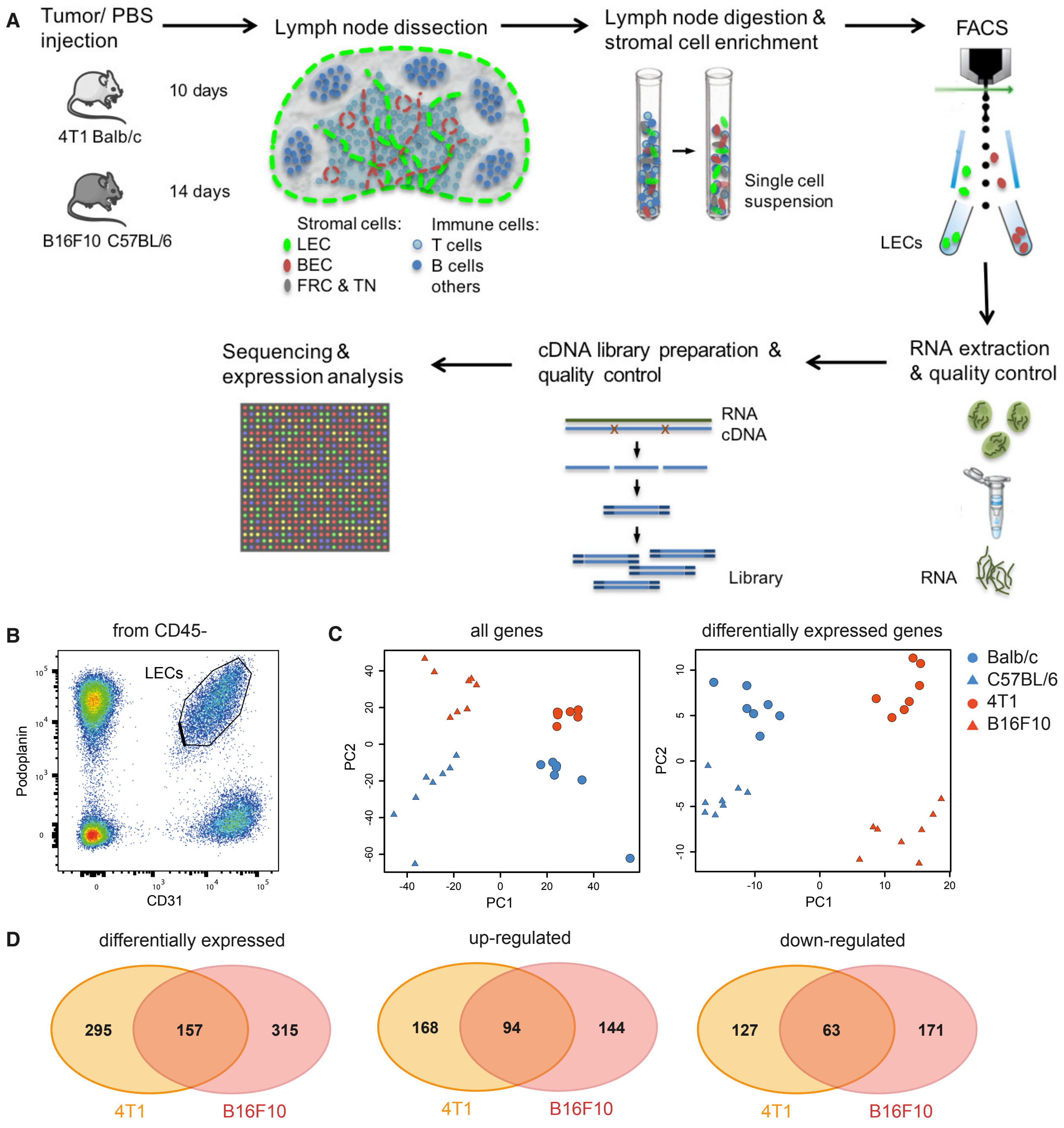

Figure 2. RNA Sequencing Reveals a Distinct Expression Profile of LECs from Tumor-Draining LNs
(A) Schematic workflow for RNA sequencing of LN LECs.
(B) Representative FACS plot showing the gating of LN LECs.
(C) Principal-component analysis (PCA) of the sequenced LEC samples based on all genes (left) and differentially expressed genes (right).
(D) Venn diagrams with the number of differentially expressed genes in LECs from tumor-draining LNs compared to naive in both tumor models.
See also Figure S3 and Table S1.

role in vascular biology because they orchestrate vessel formation, organization, stability, permeability, leukocyte transmigration, and metastasis. Genes related to cell-cell and cell-matrix adhesion were among the top differentially regulated hits in LECs of tumor-draining LNs (Figures 3A and 3B). For instance, expression of Jam3, the gene-encoding junctional adhesion molecule C (JAM-C), was significantly reduced in both tumor models (Figures 3B–3E). JAM-C is known to regulate vascular

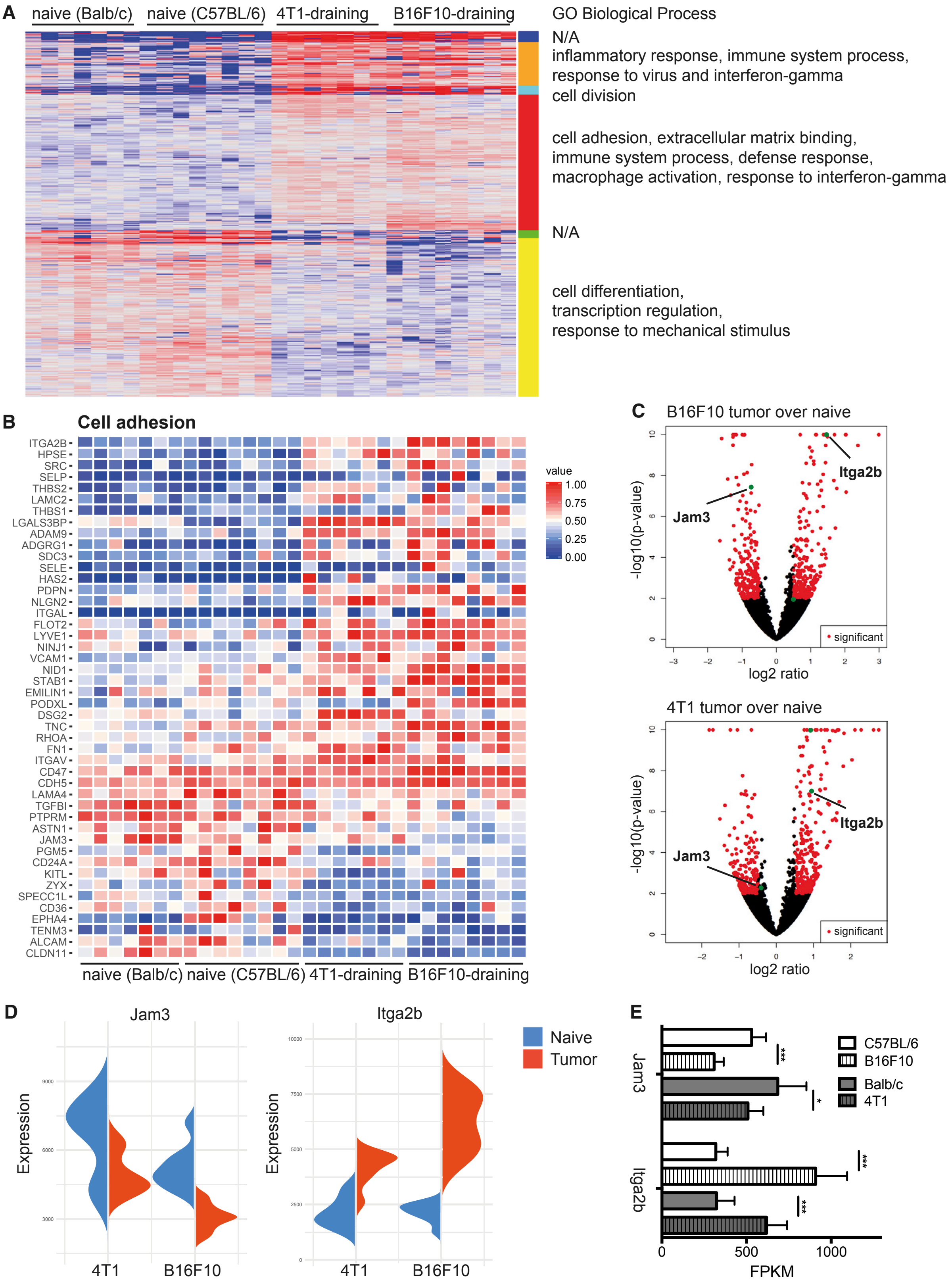

(legend on next page)

permeability, leukocyte transmigration, tumor cell interactions with endothelium, and metastasis (Fuse et al., 2007; Santoso et al., 2005; Weber et al., 2007). We confirmed JAM-C downregulation on LECs of B16F10 tumor-draining compared to naive LNs (Figure S4B), indicating that tumor-derived factors may alter the barrier function of LN LECs in this tumor model.

In conclusion, these data highlight that LECs in tumor-draining LNs respond via transcriptional alteration of pathways that are key to their activity and function, predominantly regarding immune system regulation and cell adhesion.

Integrin αIIb Is Upregulated on LECs of Tumor-Draining LNs and Mediates Adhesion of LN LECs to Fibrinogen *In Vitro*

Remarkably, RNA sequencing revealed high baseline expression of Itga2b in LN LECs, with a 2-fold increase in LECs of 4T1 tumor-draining LNs and an almost 3-fold increase in the B16F10 model (Figures 3B–3E). Itga2b codes for integrin αIIb, which is predominantly expressed by platelets, where it plays a crucial role in platelet aggregation and blood clotting. It pairs exclusively with integrin subunit β3 and binds to a variety of ligands, such as fibrinogen, fibronectin, vitronectin, and von Willebrand factor (Lefkovits et al., 1995). Recently, integrin αIIb was also shown to be expressed by a subset of LN LECs in mice and humans and to be upregulated in response to immunization (Cordeiro et al., 2016), but its function in LN LECs has not been clarified so far.

Immunofluorescence staining of LN sections revealed specific integrin αIIb staining in the lymphatic vascular network that was much stronger in tumor-draining LNs (Figure 4A). Quantification of integrin αIIb within the lymphatic area confirmed a significantly increased protein expression in LECs of both 4T1 and B16F10 tumor-draining LNs compared to their respective controls. FACS analysis of LN LECs showed a wide range of integrin αIIb expression over the whole LEC population (Figure 4B), confirming a previous report (Cordeiro et al., 2016) that integrin αIIb is only expressed by a subset of LN LECs. Although the integrin-αIIb-negative LEC subset barely expanded, the integrin-αIIb-positive population expanded massively in 4T1 tumor-draining LNs (Figure 4B). Further FACS analyses for integrin αIIb, KI67, and LYVE-1 indicated a trend toward increased proliferation of the integrin-αIIb-expressing LECs compared to integrin-αIIb-negative LECs, which however was not statistically significant (Figure S4C). We also observed integrin αIIb staining in lymphatic sinuses in human-melanoma-draining LNs (Figure 4C).

To study potential integrin αIIb functions in LECs, we isolated primary LN LECs and subjected them to functional studies *in vitro*. Using FACS analysis, we found that these cells express low levels of integrin αIIb but upregulated it in response to IFN-γ or tumor-cell-conditioned medium (CM), but not to lymphotoxin α_2/β_1 (Figure 4D). Compellingly, although both 4T1 and B16F10 CM induced an upregulation of integrin αIIb protein expression in LN LECs, B16F10 CM had a stronger effect *in vitro*, similar to what was observed on the mRNA level in tumor-draining LNs *in vivo* (Figures 3C–3E and 4D). In line with these *in vitro* results, systemic interferon (IFN)-γ blockage, but not blockade of VEGF receptor (VEGFR)-3, reduced the expression of integrin αIIb by LN LECs in 4T1-bearing mice *in vivo* (Figure 4E). Because the integrin αIIbβ3 complex in platelets strongly binds to fibrinogen, we investigated whether it might have similar ligand specificity in LN LECs. In a matrix adhesion assay, we tested the effect of integrin αIIbβ3 inhibition on binding of isolated LN LECs to fibrinogen, fibronectin, and collagen type I. Fibronectin and fibrinogen, but not collagen I, are known ligands for activated integrin αIIbβ3 on platelets. We found that blockade of integrin αIIbβ3 reduced LN LEC adhesion to fibrinogen, but not to collagen I or fibronectin (Figure 4F). Accordingly, we found a close association of LN LECs to fibrinogen and an increased presence of fibrinogen in tumor draining compared to naive LNs (Figures 4G and S4D). In comparison, integrin αIIb expression and fibrinogen deposition around lymphatic vessels in primary tumors and control skin was heterogeneous and generally weaker (Figure S4E). To further investigate the role of lymphatic integrin αIIb *in vivo*, we performed an antibody blockage experiment in 4T1-bearing mice. However, due to its effects on platelets, leading to disseminated bleeding, blockage could only be maintained for a limited time span after tumor inoculation (day 10). At this stage, no major effects on the number or proliferation rate of LN LECs were observed (Figure S4F). Taken together, these data demonstrate that tumor-associated LN LECs upregulate integrin αIIb in response to IFN-γ and suggest a role of integrin αIIb in lymphatic endothelial interaction with nodal fibrinogen.

DISCUSSION

LN swelling is common in the context of growing tumors, but the dynamics and mechanisms behind it have not been studied in detail so far. In this study, using 3D imaging of whole LNs *ex vivo*, we found that tumor-induced LN swelling is associated with a massive remodeling of the lymphatic vascular network, mediated by LEC sprouting and proliferation. These alterations are likely to be induced by lymphangiogenic factors that are drained from the primary tumor or produced by activated leukocytes, for example B cells (Angeli et al., 2006; Shrestha et al., 2010), in the tumor-draining LN because they were not detected in non-draining LNs, excluding systemic effects. Remarkably, remodeling started very early after tumor cell injection and no LN metastases were detected histologically at the studied time

Figure 3. The Expression Pattern of Cell Adhesion Molecules Is Dramatically Changed in LECs from Tumor-Draining LNs

(A) Heatmap of differentially expressed genes in tumor-draining compared to naive LN LECs. GO clusters of biological processes are indicated on the right.

(B) Heatmap of differentially expressed cell adhesion genes (GO: 0007155) in both tumor models.

(C–E) Differentially expressed genes displayed in (C) volcano plots, (D) violin plots, and (E) normalized read counts (fragments per kilobase of transcript per million mapped reads [RPKM]) in both tumor models compared to the respective naive controls.

Data are shown as mean with SD and differences were considered statistically significant when $p < 0.05$, as indicated by *$p < 0.05$, and ***$p < 0.001$. See also Figure S4 and Table S2.

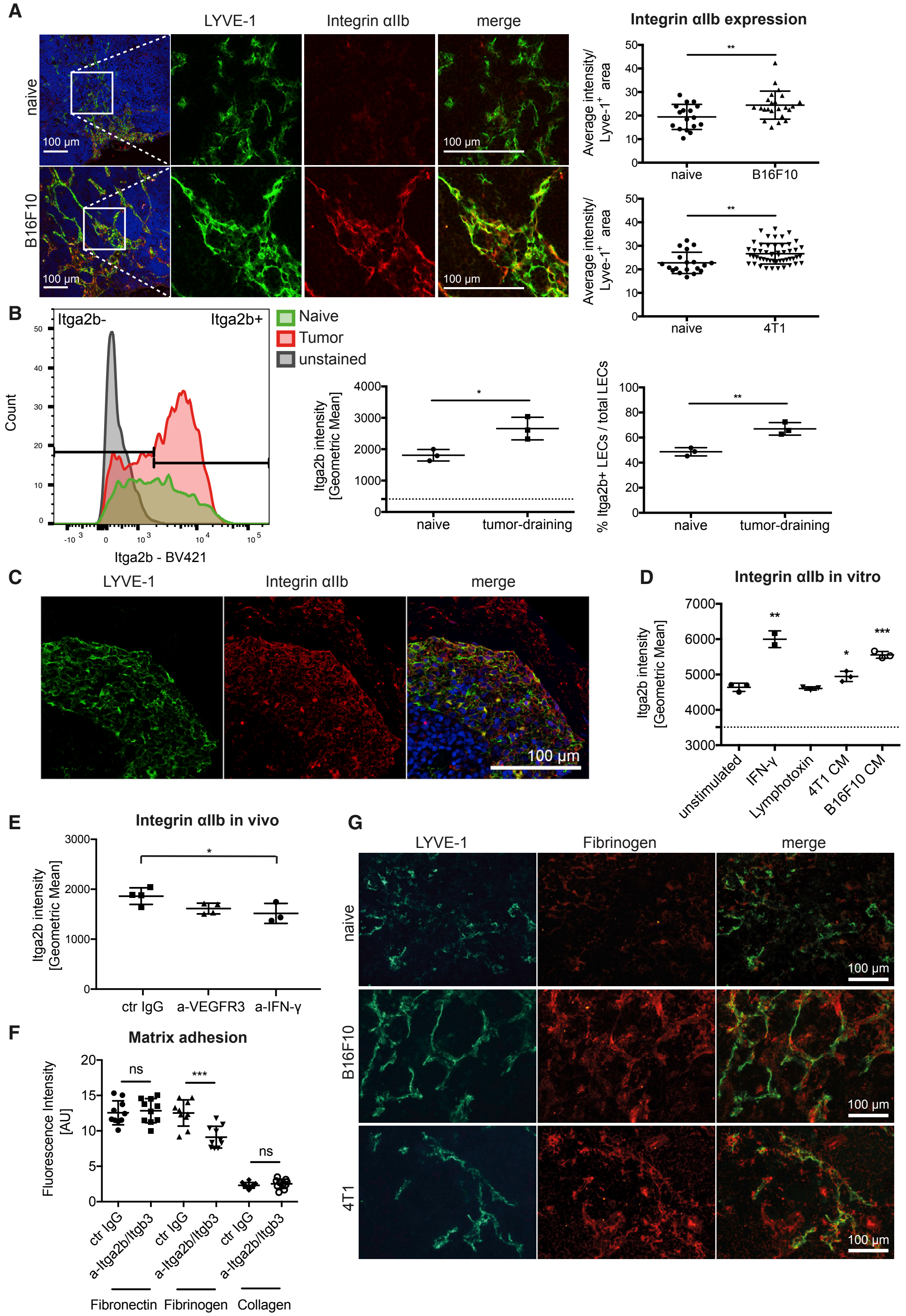

(legend on next page)

points, indicating that metastatic tumor cells are not involved in this process.

Lymphatic remodeling in advance of LN metastasis might represent the formation of a pre-metastatic, lymphovascular niche. Whereas factors such as the VEGFs, COX-2, or the heparin-binding factor midkine have been shown to mediate the formation of such niches (Hirakawa et al., 2005, 2007; Ogawa et al., 2014; Olmeda et al., 2017), the molecular profile of LECs that define them has not been described. In the present study, we performed complete RNA sequencing of LECs directly isolated from naive and tumor-draining LNs. Our data reveal that multiple cell adhesion molecules are differentially expressed in tumor-draining LN LECs. For instance, we observed downregulation of JAM-C on mRNA and protein levels in LN LECs of tumor-bearing mice. The role of JAM-C in endothelial cells is complex, and differential expression may have various consequences. High expression of JAM-C seems to increase permeability of endothelial barriers, transmigration of lymphocytes, and inflammatory leukocyte recruitment (Weber et al., 2007). Importantly, expression of JAM-C binding partners, such as JAM-C itself, was found in many human tumor lines, among them, almost all melanoma and some breast cancer lines investigated in a recent report (Klijn et al., 2015). Although the direct consequences of JAM-C downregulation in tumor-draining LN LECs are difficult to estimate, previous findings suggest that lower JAM-C levels lead to decreased vessel permeability, decreased leukocyte trafficking in the LN, and possibly decreased interaction with metastasizing tumor cells (Fuse et al., 2007; Santoso et al., 2005; Weber et al., 2007).

Interestingly, we found integrin αIIb (Itga2b) among the highest expressed and strongest upregulated genes in LN LECs and confirmed these findings on the protein level. The expression of integrin αIIb on a specific subset of LN LECs was discovered only recently, and its function in LECs has remained unknown (Cordeiro et al., 2016). We identify here that integrin αIIb mediates adhesion of LN LECs to fibrinogen *in vitro* and found colocalization of LECs with fibrinogen-rich LN areas *in vivo*. Although integrin αIIbβ3 can also bind to fibronectin, we did not observe an inhibition of LEC adhesion to fibronectin when integrin αIIbβ3 was blocked. Similarly, Cordeiro et al. (2016) found that integrin αIIb expression is not required for LEC residence in fibronectin-rich LN areas *in vivo*. This is likely due to the expression of other fibronectin-binding molecules on LECs, such as β1 integrins (Chen et al., 2012).

The extracellular matrix is greatly altered in tumor progression and plays a decisive role in lymphangiogenesis. Whereas fibrinogen is a plasma protein and usually not part of the extracellular matrix in healthy tissues, it can leak into wound areas and provide a provisional adhesive scaffold for the recruitment of cells (Chen et al., 2012). Consequently, fibrinogen was also shown to be deposited in tumor-associated extracellular matrices (Simpson-Haidaris and Rybarczyk, 2001). Given the prominent remodeling of the LN architecture during tumor progression, fibrinogen deposition in tumor-draining LNs is not unlikely to occur. Indeed, we found that fibrinogen is greatly increased in tumor-draining LNs compared to control LNs and accumulated around lymphatic sinuses. This is in line with a recent report showing that fibrinogen is very abundant in afferent lymph and is efficiently retained by draining LNs (Clement et al., 2018). Fibrinogen has been reported to promote hematogenous metastasis by shielding and protecting tumor cell emboli within the circulation (Konstantopoulos and Thomas, 2009). Interestingly, expression or plasma levels of fibrinogen also correlate with LN metastasis in mouse cancer models and in cancer patients, respectively (Adams et al., 2015; Palaj et al., 2018; Palumbo et al., 2002; Wakatsuki et al., 2017), which indicates that fibrinogen deposition in draining LNs might contribute to the formation of pre-metastatic niches, potentially via lymphatic integrin αIIb. Furthermore, integrin αIIb might also bind to fibronectin and other ligands and may thereby provide outside-in signals that promote LN LEC migration and proliferation (Durrant et al., 2017). In conclusion, expression of integrin αIIb by LN LECs could play a role in lymphangiogenesis and lymphatic network remodeling in tumor-draining LNs. Of note, inhibition of integrins has been proposed before as a means to target tumor-induced lymphangiogenesis (Chen et al., 2012). There are several integrin αIIbβ3 inhibitors (abciximab, eptifibatide, and tirofiban) that have been approved for preventative anti-thrombotic treatment to inhibit platelet aggregation and thrombus formation. However, treatment with these inhibitors might cause bleeding problems, especially in the context of leaky tumor blood vessels. Similarly, in our mouse models, inhibition of integrin αIIbβ3 using a F(ab)2 fragment could be maintained only for a short period of time, which did not suffice to detect major effects on lymphatic remodeling or subsequent metastasis. Thus, studying whether and how integrin αIIb might influence lymphatic network remodeling and tumor metastasis in tumor-draining LNs will require the generation of a lymphatic-specific knockout mouse model.

Taken together, in the present analysis, using tumor models of melanoma and breast cancer, we characterize the tumor-induced lymphatic network remodeling and describe the molecular adaptation of LECs in tumor-draining LNs. Interestingly, LECs in both

Figure 4. Integrin αIIb Is Upregulated on LECs in Tumor-Draining LNs and Mediates Adhesion of LN LECs to Fibrinogen *In Vitro*

(A) Representative images and quantification of integrin αIIb immunofluorescence stainings. Each dot represents one image; n = 8 LNs/group.

(B) FACS analysis of integrin-αIIb-positive LECs in 4T1 tumor-draining LNs. n = 3 LNs/group.

(C) Representative images of integrin αIIb expression in the subcapsular sinus of a human melanoma-draining LN.

(D) FACS analysis of integrin αIIb expression on primary murine LN LECs *in vitro* after stimulation for 24 hr. Points represent biological replicates (n = 2–3).

(E) FACS analysis of integrin αIIb expression by LN LECs *in vivo* in 4T1-bearing mice on day 10 after tumor implantation in response to VEGFR-3 and IFN-γ blockage. n = 3–4 mice/group.

(F) Matrix adhesion assay of primary murine LN LECs under integrin αIIbβ3 blockage *in vitro*. Points represent biological replicates (n = 10).

(G) LYVE-1/fibrinogen immunofluorescence stainings of naive and tumor-draining LNs.

Statistical significance was determined by the unpaired Student's t test (A, B, D [each group compared to the unstimulated control] and F) and one-way ANOVA (E). Data are shown as mean with SD and differences were considered statistically significant when $p < 0.05$, as indicated by $^{*}p < 0.05$, $^{**}p < 0.01$, and $^{***}p < 0.001$. See also Figure S4.

tumor models show an overlapping regulation of gene expression, which suggests LN LECs might not only regulate lymphangiogenesis but also the adherence and survival of metastatic tumor cells. Together, these findings indicate that LN LECs are active players in shaping tumor progression and suggest that targeting of LN LECs or specific functions of them could represent a new way to therapeutically modulate tumor metastasis.

STAR★METHODS

Detailed methods are provided in the online version of this paper and include the following:

- KEY RESOURCES TABLE
- CONTACT FOR REAGENT AND RESOURCE SHARING
- EXPERIMENTAL MODEL AND SUBJECT DETAILS
  - Mice
  - Cell lines
- METHOD DETAILS
  - Tumor models
  - 3D Light-sheet microscopy of LN whole mounts
  - Immunofluorescence staining and analysis of frozen mouse LN sections
  - Immunofluorescence staining of human LNs
  - FACS sorting and analysis of LN LECs
  - RNA extraction and sequencing of LN LECs
  - Isolation, culture and stimulation of murine LN LECs
  - Matrix adhesion assay of LN LECs
- QUANTIFICATION AND STATISTICAL ANALYSIS
- DATA AVAILABILITY

SUPPLEMENTAL INFORMATION

Supplemental Information includes four figures, two tables, and two videos and can be found with this article online at https://doi.org/10.1016/j.celrep.2018.12.002.

ACKNOWLEDGMENTS

We thank Jeannette Scholl and Carlos Ochoa Pereira for excellent technical assistance, Prof. Dr. F. Fend for the histopathology of the lymph nodes, Prof. Cornelia Halin and Simone Waldis for providing LN LECs, and Szymon Stoma for suggestions regarding image analysis. This work was supported by Swiss National Science Foundation grant 310030_166490, European Research Council grant LYVICAM, and Oncosuisse (to M.D.), and by DFG Ro 764/15-2 and SFB TRR 156 (to M.R.).

AUTHOR CONTRIBUTIONS

Conceptualization, C.D.C., L.C.D., and M.D.; Methodology, C.D.C., L.C.D., and E.R.; Formal Analysis, C.D.C., T.H., Y.H., J.A.M.-Z., and S.F.N.; Investigation, C.D.C., L.C.D., and T.H.; Writing – Original Draft, C.D.C., L.C.D., and M.D.; Resources, M.R. and M.D.; Supervision, M.D.

DECLARATION OF INTERESTS

The authors declare no competing interests.

Received: March 5, 2018
Revised: October 26, 2018
Accepted: November 30, 2018
Published: December 26, 2018

REFERENCES

Adams, G.N., Rosenfeldt, L., Frederick, M., Miller, W., Waltz, D., Kombrinck, K., McElhinney, K.E., Flick, M.J., Monia, B.P., Revenko, A.S., and Palumbo, J.S. (2015). Colon cancer growth and dissemination relies upon thrombin, stromal PAR-1, and fibrinogen. Cancer Res. *75*, 4235–4243.

Angeli, V., Ginhoux, F., Llodrà, J., Quemeneur, L., Frenette, P.S., Skobe, M., Jessberger, R., Merad, M., and Randolph, G.J. (2006). B cell-driven lymphangiogenesis in inflamed lymph nodes enhances dendritic cell mobilization. Immunity *24*, 203–215.

Broggi, M.A.S., Schmaler, M., Lagarde, N., and Rossi, S.W. (2014). Isolation of murine lymph node stromal cells. J. Vis. Exp. *90*, e51803.

Brown, M., Assen, F.P., Leithner, A., Abe, J., Schachner, H., Asfour, G., Bago-Horvath, Z., Stein, J.V., Uhrin, P., Sixt, M., and Kerjaschki, D. (2018). Lymph node blood vessels provide exit routes for metastatic tumor cell dissemination in mice. Science *359*, 1408–1411.

Chen, J., Alexander, J.S., and Orr, A.W. (2012). Integrins and their extracellular matrix ligands in lymphangiogenesis and lymph node metastasis. Int. J. Cell Biol. *2012*, 853703.

Clement, C.C., Wang, W., Dzieciatkowska, M., Cortese, M., Hansen, K.C., Becerra, A., Thangaswamy, S., Nizamutdinova, I., Moon, J.-Y., Stern, L.J., et al. (2018). Quantitative profiling of the lymph node clearance capacity. Sci. Rep. *8*, 11253.

Cordeiro, O.G., Chypre, M., Brouard, N., Rauber, S., Alloush, F., Romera-Hernandez, M., Bénézech, C., Li, Z., Eckly, A., Coles, M.C., et al. (2016). Integrin-alpha IIb identifies murine lymph node lymphatic endothelial cells responsive to RANKL. PLoS ONE *11*, e0151848.

Dadras, S.S., Lange-Asschenfeldt, B., Velasco, P., Nguyen, L., Vora, A., Muzikansky, A., Jahnke, K., Hauschild, A., Hirakawa, S., Mihm, M.C., and Detmar, M. (2005). Tumor lymphangiogenesis predicts melanoma metastasis to sentinel lymph nodes. Mod. Pathol. *18*, 1232–1242.

del Toro, R., Prahst, C., Mathivet, T., Siegfried, G., Kaminker, J.S., Larrivee, B., Breant, C., Duarte, A., Takakura, N., Fukamizu, A., et al. (2010). Identification and functional analysis of endothelial tip cell-enriched genes. Blood *116*, 4025–4033.

Dieterich, L.C., and Detmar, M. (2016). Tumor lymphangiogenesis and new drug development. Adv. Drug Deliv. Rev. *99* (*Pt B*), 148–160.

Durrant, T.N., van den Bosch, M.T., and Hers, I. (2017). Integrin $\alpha_{IIb}\beta_3$ outside-in signaling. Blood *130*, 1607–1619.

Fuse, C., Ishida, Y., Hikita, T., Asai, T., and Oku, N. (2007). Junctional adhesion molecule-C promotes metastatic potential of HT1080 human fibrosarcoma. J. Biol. Chem. *282*, 8276–8283.

García-Caballero, M., Van de Velde, M., Blacher, S., Lambert, V., Balsat, C., Erpicum, C., Durré, T., Kridelka, F., and Noël, A. (2017). Modeling pre-metastatic lymphvascular niche in the mouse ear sponge assay. Sci. Rep. *7*, 41494.

Gregory, J.L., Walter, A., Alexandre, Y.O., Hor, J.L., Liu, R., Ma, J.Z., Devi, S., Tokuda, N., Owada, Y., Mackay, L.K., et al. (2017). Infection programs sustained lymphoid stromal cell responses and shapes lymph node remodeling upon secondary challenge. Cell Rep. *18*, 406–418.

Hägerling, R., Pollmann, C., Andreas, M., Schmidt, C., Nurmi, H., Adams, R.H., Alitalo, K., Andresen, V., Schulte-Merker, S., and Kiefer, F. (2013). A novel multistep mechanism for initial lymphangiogenesis in mouse embryos based on ultramicroscopy. EMBO J. *32*, 629–644.

Harrell, M.I., Iritani, B.M., and Ruddell, A. (2007). Tumor-induced sentinel lymph node lymphangiogenesis and increased lymph flow precede melanoma metastasis. Am. J. Pathol. *170*, 774–786.

Hirakawa, S., Kodama, S., Kunstfeld, R., Kajiya, K., Brown, L.F., and Detmar, M. (2005). VEGF-A induces tumor and sentinel lymph node lymphangiogenesis and promotes lymphatic metastasis. J. Exp. Med. *201*, 1089–1099.

Hirakawa, S., Brown, L.F., Kodama, S., Paavonen, K., Alitalo, K., and Detmar, M. (2007). VEGF-C-induced lymphangiogenesis in sentinel lymph nodes promotes tumor metastasis to distant sites. Blood *109*, 1010–1017.

Hirosue, S., Vokali, E., Raghavan, V.R., Rincon-Restrepo, M., Lund, A.W., Corthésy-Henrioud, P., Capotosti, F., Halin Winter, C., Hugues, S., and Swartz, M.A. (2014). Steady-state antigen scavenging, cross-presentation, and CD8+ T cell priming: a new role for lymphatic endothelial cells. J. Immunol. *192*, 5002–5011.

Kim, M., Koh, Y.J., Kim, K.E., Koh, B.I., Nam, D.H., Alitalo, K., Kim, I., and Koh, G.Y. (2010). CXCR4 signaling regulates metastasis of chemoresistant melanoma cells by a lymphatic metastatic niche. Cancer Res. *70*, 10411–10421.

Klijn, C., Durinck, S., Stawiski, E.W., Haverty, P.M., Jiang, Z., Liu, H., Degenhardt, J., Mayba, O., Gnad, F., Liu, J., et al. (2015). A comprehensive transcriptional portrait of human cancer cell lines. Nat. Biotechnol. *33*, 306–312.

Konstantopoulos, K., and Thomas, S.N. (2009). Cancer cells in transit: the vascular interactions of tumor cells. Annu. Rev. Biomed. Eng. *11*, 177–202.

Lefkovits, J., Plow, E.F., and Topol, E.J. (1995). Platelet glycoprotein IIb/IIIa receptors in cardiovascular medicine. N. Engl. J. Med. *332*, 1553–1559.

Li, Y., Reader, J.C., Ma, X., Kundu, N., Kochel, T., and Fulton, A.M. (2015). Divergent roles of CXCR3 isoforms in promoting cancer stem-like cell survival and metastasis. Breast Cancer Res. Treat. *149*, 403–415.

Malhotra, D., Fletcher, A.L., Astarita, J., Lukacs-Kornek, V., Tayalia, P., Gonzalez, S.F., Elpek, K.G., Chang, S.K., Knoblich, K., Hemler, M.E., et al.; Immunological Genome Project Consortium (2012). Transcriptional profiling of stroma from inflamed and resting lymph nodes defines immunological hallmarks. Nat. Immunol. *13*, 499–510.

Mandriota, S.J., Jussila, L., Jeltsch, M., Compagni, A., Baetens, D., Prevo, R., Banerji, S., Huarte, J., Montesano, R., Jackson, D.G., et al. (2001). Vascular endothelial growth factor-C-mediated lymphangiogenesis promotes tumour metastasis. EMBO J. *20*, 672–682.

Müller, A., Homey, B., Soto, H., Ge, N., Catron, D., Buchanan, M.E., McClanahan, T., Murphy, E., Yuan, W., Wagner, S.N., et al. (2001). Involvement of chemokine receptors in breast cancer metastasis. Nature *410*, 50–56.

Ogawa, F., Amano, H., Eshima, K., Ito, Y., Matsui, Y., Hosono, K., Kitasato, H., Iyoda, A., Iwabuchi, K., Kumagai, Y., et al. (2014). Prostanoid induces premetastatic niche in regional lymph nodes. J. Clin. Invest. *124*, 4882–4894.

Olmeda, D., Cerezo-Wallis, D., Riveiro-Falkenbach, E., Pennacchi, P.C., Contreras-Alcalde, M., Ibarz, N., Cifdaloz, M., Catena, X., Calvo, T.G., Cañón, E., et al. (2017). Whole-body imaging of lymphovascular niches identifies premetastatic roles of midkine. Nature *546*, 676–680.

Palaj, J., Kečkéš, Š., Marek, V., Dyttert, D., Waczulíková, I., and Durdík, Š. (2018). Fibrinogen levels are associated with lymph node involvement and overall survival in gastric cancer patients. Anticancer Res. *38*, 1097–1104.

Palumbo, J.S., Potter, J.M., Kaplan, L.S., Talmage, K., Jackson, D.G., and Degen, J.L. (2002). Spontaneous hematogenous and lymphatic metastasis, but not primary tumor growth or angiogenesis, is diminished in fibrinogen-deficient mice. Cancer Res. *62*, 6966–6972.

Pastushenko, I., Van den Eynden, G.G., Vicente-Arregui, S., Prieto-Torres, L., Alvarez-Alegret, R., Querol, I., Dirix, L.Y., Carapeto, F.J., Vermeulen, P.B., and Van Laere, S.J. (2016). Increased angiogenesis and lymphangiogenesis in metastatic sentinel lymph nodes is associated with nonsentinel lymph node involvement and distant metastasis in patients with melanoma. Am. J. Dermatopathol. *38*, 338–346.

Pereira, E.R., Kedrin, D., Seano, G., Gautier, O., Meijer, E.F.J., Jones, D., Chin, S.-M., Kitahara, S., Bouta, E.M., Chang, J., et al. (2018). Lymph node metastases can invade local blood vessels, exit the node, and colonize distant organs in mice. Science *359*, 1403–1407.

Qian, C.N., Berghuis, B., Tsarfaty, G., Bruch, M., Kort, E.J., Ditlev, J., Tsarfaty, I., Hudson, E., Jackson, D.G., Petillo, D., et al. (2006). Preparing the "soil": the primary tumor induces vasculature reorganization in the sentinel lymph node before the arrival of metastatic cancer cells. Cancer Res. *66*, 10365–10376.

Riedel, A., Shorthouse, D., Haas, L., Hall, B.A., and Shields, J. (2016). Tumor-induced stromal reprogramming drives lymph node transformation. Nat. Immunol. *17*, 1118–1127.

Rouhani, S.J., Eccles, J.D., Tewalt, E.F., and Engelhard, V.H. (2014). Regulation of T-cell tolerance by lymphatic endothelial cells. J. Clin. Cell. Immunol. *5*, 242.

Santoso, S., Orlova, V.V., Song, K., Sachs, U.J., Andrei-Selmer, C.L., and Chavakis, T. (2005). The homophilic binding of junctional adhesion molecule-C mediates tumor cell-endothelial cell interactions. J. Biol. Chem. *280*, 36326–36333.

Schindelin, J., Arganda-Carreras, I., Frise, E., Kaynig, V., Longair, M., Pietzsch, T., Preibisch, S., Rueden, C., Saalfeld, S., Schmid, B., et al. (2012). Fiji: an open-source platform for biological-image analysis. Nat. Methods *9*, 676–682.

Schneider, C.A., Rasband, W.S., and Eliceiri, K.W. (2012). NIH Image to ImageJ: 25 years of image analysis. Nat. Methods *9*, 671–675.

Shrestha, B., Hashiguchi, T., Ito, T., Miura, N., Takenouchi, K., Oyama, Y., Kawahara, K., Tancharoen, S., Ki-I, Y., Arimura, N., et al. (2010). B cell-derived vascular endothelial growth factor A promotes lymphangiogenesis and high endothelial venule expansion in lymph nodes. J. Immunol. *184*, 4819–4826.

Simpson-Haidaris, P.J., and Rybarczyk, B. (2001). Tumors and fibrinogen. The role of fibrinogen as an extracellular matrix protein. Ann. N Y Acad. Sci. *936*, 406–425.

Skobe, M., Hawighorst, T., Jackson, D.G., Prevo, R., Janes, L., Velasco, P., Riccardi, L., Alitalo, K., Claffey, K., and Detmar, M. (2001). Induction of tumor lymphangiogenesis by VEGF-C promotes breast cancer metastasis. Nat. Med. *7*, 192–198.

Stacker, S.A., Caesar, C., Baldwin, M.E., Thornton, G.E., Williams, R.A., Prevo, R., Jackson, D.G., Nishikawa, S., Kubo, H., and Achen, M.G. (2001). VEGF-D promotes the metastatic spread of tumor cells via the lymphatics. Nat. Med. *7*, 186–191.

Stacker, S.A., Williams, S.P., Karnezis, T., Shayan, R., Fox, S.B., and Achen, M.G. (2014). Lymphangiogenesis and lymphatic vessel remodelling in cancer. Nat. Rev. Cancer *14*, 159–172.

Strasser, G.A., Kaminker, J.S., and Tessier-Lavigne, M. (2010). Microarray analysis of retinal endothelial tip cells identifies CXCR4 as a mediator of tip cell morphology and branching. Blood *115*, 5102–5110.

Subramanian, A., Tamayo, P., Mootha, V.K., Mukherjee, S., Ebert, B.L., Gillette, M.A., Paulovich, A., Pomeroy, S.L., Golub, T.R., Lander, E.S., and Mesirov, J.P. (2005). Gene set enrichment analysis: a knowledge-based approach for interpreting genome-wide expression profiles. Proc. Natl. Acad. Sci. USA *102*, 15545–15550.

Van den Eynden, G.G., Vandenberghe, M.K., van Dam, P.J.H., Colpaert, C.G., van Dam, P., Dirix, L.Y., Vermeulen, P.B., and Van Marck, E.A. (2007). Increased sentinel lymph node lymphangiogenesis is associated with nonsentinel axillary lymph node involvement in breast cancer patients with a positive sentinel node. Clin. Cancer Res. *13*, 5391–5397.

Wakatsuki, K., Matsumoto, S., Migita, K., Ito, M., Kunishige, T., Nakade, H., Nakatani, M., Kitano, M., and Sho, M. (2017). Preoperative plasma fibrinogen is associated with lymph node metastasis and predicts prognosis in resectable esophageal cancer. World J. Surg. *41*, 2068–2077.

Weber, C., Fraemohs, L., and Dejana, E. (2007). The role of junctional adhesion molecules in vascular inflammation. Nat. Rev. Immunol. *7*, 467–477.

Wiley, H.E., Gonzalez, E.B., Maki, W., Wu, M.T., and Hwang, S.T. (2001). Expression of CC chemokine receptor-7 and regional lymph node metastasis of B16 murine melanoma. J. Natl. Cancer Inst. *93*, 1638–1643.

Williams, S.P., Odell, A.F., Karnezis, T., Farnsworth, R.H., Gould, C.M., Li, J., Paquet-Fifield, S., Harris, N.C., Walter, A., Gregory, J.L., et al. (2017). Genome-wide functional analysis reveals central signaling regulators of lymphatic endothelial cell migration and remodeling. Sci. Signal. *10*, eaal2987.

STAR★METHODS

KEY RESOURCES TABLE

REAGENT or RESOURCE	SOURCE	IDENTIFIER
Antibodies		
Rat monoclonal anti-VEGFR3 clone mF4-31C1	ImClone Systems / Eli Lilly	N/A
Rat monoclonal anti-IFN-γ clone R4-6A2	BioXCell	BE0054, RRID:AB_1107692
Rat control IgG	Sigma Aldrich	I4131, RRID:AB_1163627
Rat monoclonal anti-Itga2b clone Leo.H4	Emfret	M021-0
Rat monoclonal anti-KLH clone LTF-2	BioXCell	BE0090, RRID:AB_1107780
Goat anti-Lyve-1	R&D	AF2125, RRID:AB_2297188
Rabbit anti-Lyve-1	AngioBio	11-034
Rat monoclonal anti-Lyve-1 clone 4D17	ReliaTech	103-M130
Goat anti-Prox1	R&D	AF2727, RRID:AB_2170716
Rat monoclonal anti-Ki67 clone TEC-3	Dako	M7249, RRID:AB_2250503
Rat monoclonal anti-CD41 clone MWReg30	BD	553847, RRID:AB_395084
Rabbit anti-Jam-C	Prof. Beat Imhof, University of Geneva	N/A
Rabbit anti-Fibrinogen	Dako	A0080, RRID:AB_578481
Rabbit anti-Cytokeratin	Dako	Z0622, RRID:AB_2650434
Rabbit anti-GP100	abcam	ab137078, RRID:AB_2732921
Goat anti-Lyve-1 Biotin	R&D	BAF2089, RRID:AB_356247
Rabbit anti-CD41	Sigma Aldrich	HPA031168, RRID:AB_10664706
Mouse monoclonal anti-CD45.2 FITC clone 104	BD	553772, RRID:AB_395041
Rat monoclonal anti-CD45 APC-Cy7 clone 30-F11	BioLegend	103116, RRID:AB_312981
Rat monoclonal anti-CD31 APC clone MEC13.3	BD	551262, RRID:AB_398497
Hamster monoclonal anti-podoplanin PE clone 8.1.1	eBioscience	12-5381-80, RRID:AB_1907440
Rat monoclonal anti-CD41 BV421 clone MWReg30	BioLegend	133911, RRID:AB_10960744
Goat anti-GPNMB	R&D	AF2330, RRID:AB_2112934
Rat monoclonal anti-Ki67 eFluor450 clone SolA15	eBioscience	48-5698-82, RRID:AB_11149124
Biological Samples		
Human melanoma-draining lymph node sections	This paper	N/A
Chemicals, Peptides, and Recombinant Proteins		
Mouse IFN-γ	Peprotech	315-05
Mouse lymphotoxin α_2/β_1	R&D	1008-LY
Deposited Data		
RNA sequencing data	This paper	ENA: PRJEB22969
Experimental Models: Cell Lines		
4T1-luc2	Caliper Life Sciences	124087, RRID:CVCL_L899
B16F10	ATCC	CRL-6475, RRID:CVCL_0159
Experimental Models: Organisms/Strains		
Balb/cByJRj wildtype mice	Janvier	N/A
C57BL/6JRj wildtype mice	Janvier	N/A

CONTACT FOR REAGENT AND RESOURCE SHARING

Further information and requests for resources and reagents should be directed to and will be fulfilled by the Lead Contact, Michael Detmar (michael.detmar@pharma.ethz.ch).

EXPERIMENTAL MODEL AND SUBJECT DETAILS

Mice

Female Balb/cByJRj (referred to as BALB/c) mice and C57BL/6JRj (referred to as C57BL/6) mice were purchased from Janvier and housed in an SOPF facility with free access to food and drinking water. Mice were used for tumor studies at an age of 10-12 weeks (BALB/c) or 8-10 weeks (C57BL/6). All experiments were approved by the Cantonal Veterinary Office Zurich (license numbers ZH011/12, ZH012/15, and ZH005/18).

Cell lines

4T1 breast carcinoma cells expressing luc2 (Caliper Life Sciences) were cultured in DMEM supplemented with L-Glutamine and 10% FBS (all GIBCO). B16F10 cells (ATCC) were cultured in DMEM supplemented with GlutaMax, Pyruvate and 10% FBS (all GIBCO). All cell lines were maintained at 37°C in a humified incubator with 5% CO_2, and were routinely tested for mycoplasma contamination (Mycoscope, Genlantis).

METHOD DETAILS

Tumor models

BALB/c mice were injected subcutaneously with $1x10^5$ 4T1 cells in 50 μl PBS (or PBS alone as control) into the 4th mammary fat pad and tumors were grown for 10 days (for RNA sequencing) or 20 days, unless indicated otherwise. C57BL/6 mice were injected intradermally with $1x10^5$ B16F10 cells (or PBS alone as control) into the flank and tumors were grown for 14 days, unless indicated otherwise.

In some experiments, mice were treated intraperitoneally on day 2, 5 and 8 after tumor inoculation with rat anti-mouse VEGFR3 (mF4-31C1, Imclone Systems Inc. / Eli Lilly, 800 μg / injection), rat anti-mouse IFN-γ (R4-6A2, BioXCell, 300 μg / injection) or control rat IgG (Sigma Aldrich, 800 μg / injection).

For integrin αIIb blocking experiments, F(ab)2 fragments were generated from a rat IgG2b control antibody (LTF-2, BioXCell) and an integrin αIIb blocking antibody (Leo.H4, EMFRET) using the Pierce F(ab)2 generation kit according to the manufacturer's instructions (Thermo Fisher). The resulting F(ab)2 fragments were sterile filtered and endotoxin-cleared using Pierce Detoxi-Gel columns (Thermo Fisher). Biologic activity was confirmed using a tail bleeding test (data not shown). 4T1 tumor-bearing mice were treated from day 4 to day 9 by intraperitoneal injection of 1 μg / g body weight of the F(ab)2 fragments and euthanized at day 10.

3D Light-sheet microscopy of LN whole mounts

Whole inguinal LNs were stained by immunofluorescence and optically cleared for light-sheet microscopy as previously described (Hägerling et al., 2013). In brief, LNs (naive or tumor-draining) were fixed in 4% paraformaldehyde for 2 hours and permeabilized for 2 days using 0.5% Triton X-100 in PBS. The LNs were then incubated in blocking solution (1% bovine serum albumin (BSA), 0.1% Tween20, 0.03% NaN_3 in PBS) for 2 days before the primary anti-LYVE-1 antibody (R&D AF2125 or AngioBio 11-034, 1:200) was added in blocking solution for 7 days at 4°C. Subsequently, LNs were washed, incubated for 7 days at 4°C with the secondary antibody (donkey anti-rabbit or anti-goat Alexa 488, Invitrogen, 1:200) in blocking solution, and washed again. For optical clearing of the tissue, LNs were embedded in 1% agarose (Ultrapure LMP Agarose, Invitrogen) in water. Following dehydration with a series of 50%, 70%, 95%, and 2x 100% methanol, the tissue was incubated in BABB (1:2 benzyl alcohol in benzyl benzoate, both Sigma Aldrich) for at least 2 days until optically cleared. 3D image stacks were taken with a light-sheet microscope (LaVision BioTech) with a 2x or a 6.3x objective and processed using Imaris (Bitplane) or Fiji (ImageJ, (Schindelin et al., 2012)) software.

Immunofluorescence staining and analysis of frozen mouse LN sections

Inguinal LNs were embedded in OCT (Tissue-Tek) and frozen at −80°C. 7 μm thick tissue sections were fixed with acetone and 80% methanol, washed, and incubated with blocking solution (5% donkey serum, 1% BSA, 0.05% NaN_3, 0.1% Triton X-100 in PBS) for 1 hour. Tissue sections were incubated with primary antibodies in blocking solution at 4°C overnight. Primary antibodies against LYVE-1 (AngioBio 11-034, 1:600 or R&D AF2125, 1:100 or ReliaTech 103-M130, 1:100), Prox-1 (R&D AF2727, 1:100), Ki67 (Dako M7249, 1:200), CD41/ integrin αIIb (BD 553847, 1:50), JAM-C (kindly provided by Prof. Beat Imhof, Université de Genève, 1:500), fibrinogen (Dako A0080, 1:100), pan-keratin/ wide spectrum Cytokeratin (Dako Z0622, 1:500), and melanoma gp100 (Abcam ab137078, 1:100) were used. After washing, sections were incubated with appropriate secondary antibodies (donkey anti-rabbit Alexa 350, donkey anti-rabbit, anti-goat or anti-rat Alexa 488, donkey anti-rabbit, anti-goat or anti-rat Alexa 594, donkey anti-rabbit Alexa 647; all Invitrogen, 1:200) for 30 min, washed, and mounted using Mowiol. Hoechst 33342 was used for nuclear staining. Images were acquired on a fluorescence microscope (Axioskop 2 mot plus) or an LSM 780 inverted confocal microscope (both Carl Zeiss).

Quantification of LEC proliferation was done using the Colocalization Analysis Plugin in ImageJ (Schneider et al., 2012). Data are shown as pooled data from 2 individual studies with a total of n = 8 mice per time point. Proliferation is expressed as percent of proliferating LECs ($Ki67^+$) of total Prox1-positive LECs.

JAM-C and integrin αIIb staining was quantified with custom code written in MATLAB (MathWorks) and is shown as average pixel intensity over the LYVE-1 positive area per image. In brief, segmentation of the LYVE-1 positive area was done using the median filter to remove local noise and the Kittler thresholding algorithm. Average intensity of JAM-C and integrin αIIb staining within the obtained segmentation masks was measured in n = 8 individual LNs per condition.

Immunofluorescence staining of human LNs

Tumor draining LNs from melanoma patients were collected at the Department of Dermatology, Tübingen, Germany. The presence of metastatic cells was determined by a board-certified pathologist. For stainings, formalin-fixed, paraffin-embedded tissue sections were deparaffinized and subjected to antigen retrieval in citrate buffer. Primary antibodies against LYVE-1 (biotinylated, R&D BAF2089, 1:50) and CD41/ integrin αIIb (Sigma HPA31168, 1:200) diluted in blocking solution were incubated over night at 4°C. After washing, slides were incubated with Streptavidin Alexa488 (1:200) and donkey anti-rabbit Alexa 594 (1:200) before mounting with Mowiol. Images were acquired on an LSM 780 inverted confocal microscope (Carl Zeiss).

FACS sorting and analysis of LN LECs

For FACS sorting, 4T1 tumors were grown until day 10 and B16F10 tumors until day 14 before LN dissection. 4 naive inguinal LNs or 2 tumor-draining inguinal LNs were pooled per sample to obtain sufficient cell numbers. For each group, n = 7 (4T1 and BALB/c) or n = 8 (B16F10 and C57BL/6) samples were sorted. For FACS analysis, single LNs were stained and analyzed.

For sorting and analysis of LN stromal cells, LN suspensions were enriched for the stromal cell populations using a modified published technique (Broggi et al., 2014). In brief, LNs were dissected and the capsule was ruptured in cold basic medium (2%FCS, 1.2 mM $CaCl_2$, in DMEM medium, GIBCO) using needles. After a pre-digestion in 1 mg/ml collagenase IV (GIBCO) and 40 μg/ml DNase I (Roche) in basic medium for 20 min at 37°C, the non-stromal cell supernatant was removed to enrich for LN stromal cells. The remaining LN fragments were digested with 3.5 mg/ml collagenase IV and 40 μg/ml DNase I in basic medium for 15 min at 37°C. Subsequently, LN fragments were mechanically disaggregated using an automated multichannel pipette (Eppendorf) and 5 mM EDTA was added to ensure maintenance of single cell suspension. Basic medium was added before the cell suspension was filtered through a 40 μm cell strainer (BD).

For tumor cell analysis, LNs and tumors were minced and digested with 4 mg/ml collagenase IV (GIBCO) and 40 μg/ml DNase I (Roche) in basic medium for 25 min at 37°C. LN samples were directly filtered through a 40 μm cell strainer (BD). Tumor samples were first filtered through a 70 μm cell strainer (BD), subjected to ACK lysis (Pharm Lyse, BD 555899), and also filtered through a 40 μm cell strainer (BD).

Cells were stained with fluorescent antibodies for 20 min on ice. Antibodies used were CD45.2 – FITC (BD 553772, 1:100) or CD45 – APC-Cy7 (BioLegend 103116, 1:400), CD31 – APC (BD 551262, 1:200), podoplanin – PE (8.1.1 eBioscience, 1:200), and CD41/integrin αIIb – BV421 (BioLegend 133911, 1:100). GPNMB was stained with an unconjugated primary antibody (R&D AF2330, 1:100) in combination with an Alexa 488-conjugated secondary antibody (Invitrogen A-11055, 1:200). Intracellular staining for Ki67 – eFluor450 (SOLA15 eBioscience, 1:200) was done using the Foxp3 intracellular staining kit (eBioscience) according to the instructions. After washing, cells were resuspended in FACS buffer (1% FBS, 2 mM EDTA in PBS) for sorting or acquisition. Live/dead staining was done either directly before acquisition (7-AAD, BioLegend) or together with the antibody incubation (Zombie NIR, BioLegend).

For RNA sequencing, LN LECs were sorted for high purity on a FACS Aria II (BD). Cells were sorted directly into RNA lysis buffer (RLT Plus, QIAGEN), vortexed, and immediately frozen at −80°C until RNA extraction. FACS acquisition was done on a LSRFortessa (BD).

RNA extraction and sequencing of LN LECs

RNA was extracted and genomic DNA was eliminated from sorted LN LECs using the RNeasy Plus Micro kit (QIAGEN). RNA quantity and quality were assessed using a Bioanalyzer (Agilent). cDNA libraries were generated from high quality RNA samples using the Ovation Single Cell RNA-Seq System (NuGEN) and cDNA library quality was tested on a TapeStation (Agilent). RNA sequencing and differential expression analysis was performed by the Functional Genomics Center Zurich (FGCZ). For each group, n = 7 (4T1 and BALB/c) or n = 8 (B16F10 and C57BL/6) individual samples were sequenced using an Illumina Hiseq System. Sequencing depth was at least 3 mio read counts per sample. Differential gene expression analysis (individually for both tumor models and pooled for both) was done using DESeq2. Threshold for differential expression was defined as log2 fold change > 0.5, p value < 0.01. Principal component analysis (PCA), Venn diagrams (‘VennDiagram’ package), violin plots and gene expression heatmaps (‘ggplot2’ package) were generated in R (v3.4.0). For heatmaps, gene expression was normalized and is shown as percent of maximum gene expression for each gene. The top 50 differentially expressed genes were furthermore compared to previously published gene expression datasets of LN LECs in pathological conditions. Gene expression data of LN LECs from day 6 after HSV infection (Gregory et al., 2017) were used as published. Gene expression of LN LECs during inflammation induced by ovalbumin injection into mice after adoptive transfer of OT-1 T cells (Malhotra et al., 2012) was retrieved from GEO (GSE15907) and was re-analyzed using Geo2R. A log_2FC of ± 1 and an FDR < 0.05 was used to select differentially expressed genes. Gene set enrichment analysis (GSEA) comparing tumor-draining with naive LN LECs was performed on previously documented gene signatures of endothelial sprouting tip cells (del Toro et al., 2010; Strasser et al., 2010) using GSEA software provided by the Broad Institute (Subramanian et al., 2005). P values were estimated by 1000 permutations.

Isolation, culture and stimulation of murine LN LECs

LN LECs were isolated as previously described (Hirosue et al., 2014). In brief, LNs were digested with 0.25 mg/ml Liberase DH (Roche) and 200 U/ml DNase I (Sigma Aldrich) in RPMI medium (GIBCO) for 1 hour at 37°C. LN cell suspensions were filtered and plated in LN LEC medium (MEM alpha medium supplemented with 1x penicillin/streptomycin, 10% FBS, and 1x L-glutamine, all from GIBCO) on cell culture dishes pre-coated with 10 μg/ml fibronectin (Chemicon) and 10 μg/ml collagen (PureCol, Advanced BioMatrix). Cells were grown to confluency with regular exchange of the medium to remove non-adherent cells and enrich for LECs. Subsequently, cells were detached with Accutase (Biological Industries) and endothelial cells were positively selected with $CD31^+$ microbeads (Miltenyi Biotech). LEC purity was checked by FACS analysis and LN LECs were cultured and used for *in vitro* assays up to passage 4 after isolation.

LECs were treated with 100 ng/ml murine interferon (IFN)-γ (Peprotech), 100 ng/ml murine lymphotoxin α_2/β_1 (LT, R&D), or with 4T1 or B16F10 tumor cell conditioned media (CM) for 24 hours before they were used for assays. For 4T1 and B16F10 CM, tumor cells were grown to 80% confluency, washed, and incubated in LN LEC medium for 24 hours. Conditioned medium was then collected, sterile filtered and stored at −20°C.

Matrix adhesion assay of LN LECs

Isolated LN LECs were grown to near-confluency and incubated with IFN-γ for 24 hours to increase integrin αIIb expression. Subsequently, LECs were stained with 6 μM calcein (Invitrogen) in PBS for 10 min at 37°C. Cells were then washed with PBS and incubated with medium for 3 hours to remove excess dye, before they were detached by mild trypsinization. LECs were then incubated with 10 μg/ml integrin αIIbβ3 blocking antibody (Leo.H4, EMFRET) or control rat IgG (Sigma Aldrich I4131) in full medium for 30 min. 96-well-plates were coated with 10 μg/ml fibronectin (Chemicon), fibrinogen (abcam), or collagen (PureCol, Advanced BioMatrix) for 30 min at RT and subsequently blocked with 0.1% BSA/PBS for 30 min at 37°C before $1x10^4$ LECs/ well were added in media containing the integrin αIIbβ3 blocking antibody or the IgG control. Cells were allowed to adhere to the matrix for 1 hour at 37°C, then washed twice with PBS. Adherent cells were detected at 485 nm excitation and 538 nm emission wavelength using a fluorescence plate reader (SpectraMax, Molecular Devices).

QUANTIFICATION AND STATISTICAL ANALYSIS

Statistical analysis was performed with GraphPad Prism 6.0 software using the unpaired Student's t test, one- or two-way ANOVA, as indicated in the figure legends. Data are shown as mean with standard deviation and differences were considered statistically significant when $p < 0.05$, as indicated by asterisks with $p < 0.05$ (*), $p < 0.01$ (**) and $p < 0.001$ (***).

DATA AVAILABILITY

The accession number for the RNA sequencing data reported in this paper is ENA: PRJEB22969.

Cell Reports, Volume *25*

Supplemental Information

Mechanisms of Tumor-Induced Lymphovascular Niche Formation in Draining Lymph Nodes

Catharina D. Commerford, Lothar C. Dieterich, Yuliang He, Tanja Hell, Javier A. Montoya-Zegarra, Simon F. Noerrelykke, Erica Russo, Martin Röcken, and Michael Detmar

Figure S1:

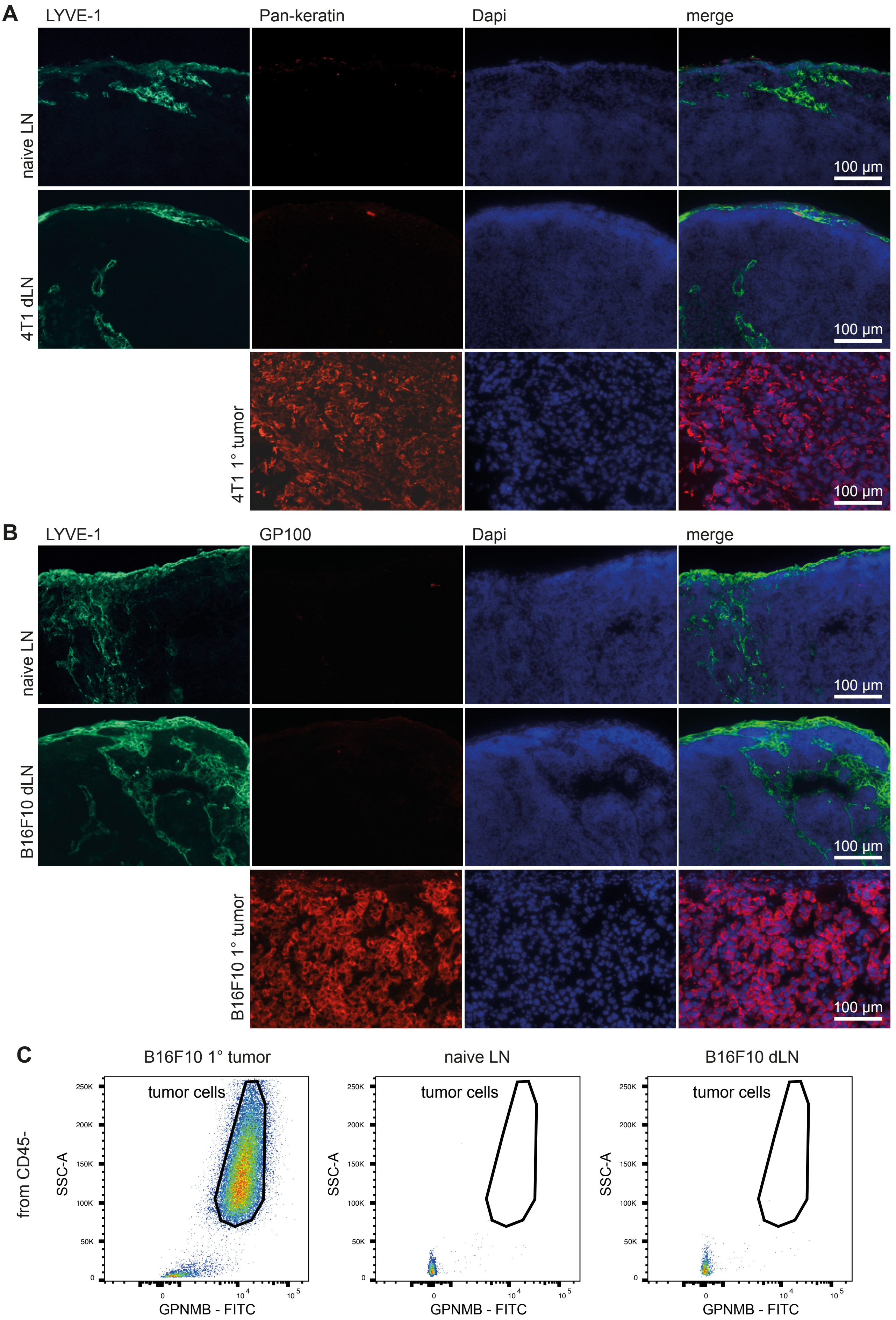
A
LYVE-1
Pan-keratin
Dapi
merge
naive LN
4T1 dLN
4T1 1° tumor
100 µm
B
LYVE-1
GP100
Dapi
merge
naive LN
B16F10 dLN
B16F10 1° tumor
100 µm
C
B16F10 1° tumor
naive LN
B16F10 dLN
from CD45-
tumor cells
SSC-A
GPNMB - FITC
250K
200K
150K
100K
50K
0
10^4
10^5

Figure S1. No metastatic tumor cells in 4T1 and B16F10 tumor-draining LNs. Related to Figure 1.
(A and B) Representative immunofluorescence images of naïve LNs, tumor-draining LNs, and primary tumors stained for (A) pan-keratin or (B) the melanoma marker gp100. The pan-keratin staining shows some artefacts in both naïve, as well as 4T1 tumor-draining LNs. (C) Representative FACS plots showing GPNMB+ melanoma cells in B16F10 primary tumors but not in naïve or tumor-draining LNs. Cells were pre-gated on single, living, CD45- cells.

Figure S2:

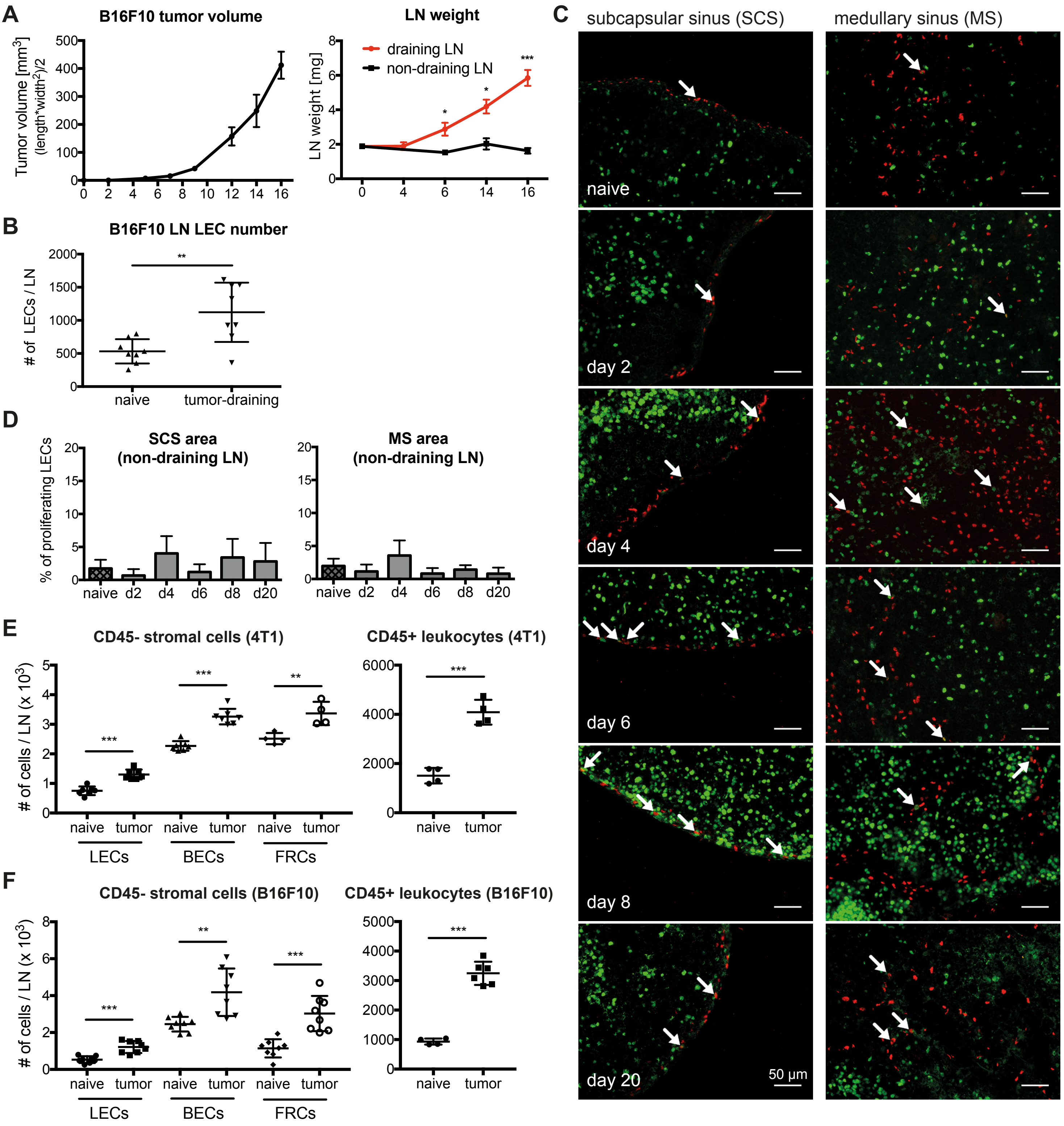
A
B16F10 tumor volume
Tumor volume [mm3] (length*width2)/2
LN weight
draining LN
non-draining LN
LN weight [mg]
B
B16F10 LN LEC number
# of LECs / LN
naive
tumor-draining
C
subcapsular sinus (SCS)
medullary sinus (MS)
naive
day 2
day 4
day 6
day 8
day 20
50 µm
D
SCS area (non-draining LN)
MS area (non-draining LN)
% of proliferating LECs
naive d2 d4 d6 d8 d20
E
CD45- stromal cells (4T1)
CD45+ leukocytes (4T1)
# of cells / LN (x 103)
naive tumor naive tumor naive tumor
LECs BECs FRCs
F
CD45- stromal cells (B16F10)
CD45+ leukocytes (B16F10)

Figure S2. LN weight and LEC proliferation increase over time in tumor-draining LNs. Related to Figure 1.

(A) B16F10 primary tumor growth assessed by caliper measurements *in vivo* and *ex vivo* inguinal LN weight.
(B) FACS quantification of LEC numbers in naïve and B16F10 tumor-draining LNs at day 14.
(C) Representative images of the subcapsular and medullary sinus (SCS & MS) of 4T1 tumor-draining LNs at different time points stained for Prox1 (red) and Ki67 (green), with arrows indicating proliferating LECs.
(D) Quantification of proliferation in the SCS & MS of non-draining contralateral inguinal LNs in 4T1 mice.
(E and F) FACS quantification shows increased LEC, BEC, FRC and leukocyte numbers in (E) 4T1 and (F) B16F10 tumor-draining LNs compared to the respective naïve control LNs. Statistical significance was determined with the unpaired student t-test (A, B, E, F) or one-way ANOVA (D).

Figure S3:

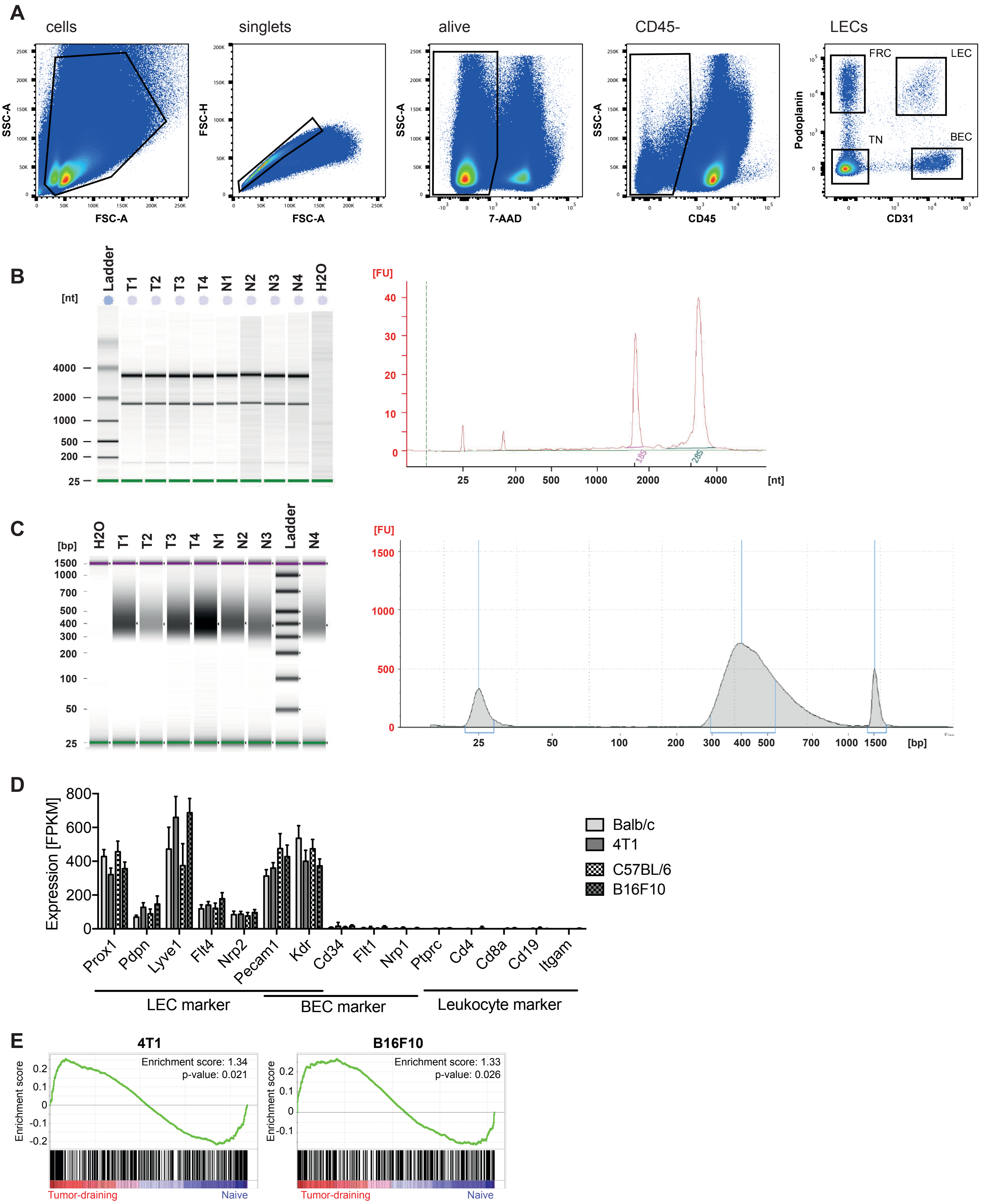
A
cells
singlets
alive
CD45-
LECs
SSC-A
FSC-A
FSC-H
7-AAD
CD45
Podoplanin
CD31
FRC
LEC
TN
BEC
B
Ladder
T1
T2
T3
T4
N1
N2
N3
N4
H2O
[nt]
4000
2000
1000
500
200
25
[FU]
40
30
20
10
0
18S
28S
[nt]
C
[bp]
1500
1000
700
500
400
300
200
100
50
25
[FU]
[bp]
D
Expression [FPKM]
800
600
400
200
0
Prox1
Pdpn
Lyve1
Flt4
Nrp2
Pecam1
Kdr
Cd34
Flt1
Nrp1
Ptprc
Cd4
Cd8a
Cd19
Itgam
LEC marker
BEC marker
Leukocyte marker
Balb/c
4T1
C57BL/6
B16F10
E
4T1
B16F10
Enrichment score
Enrichment score: 1.34
p-value: 0.021
Enrichment score: 1.33
p-value: 0.026
Tumor-draining
Naive

Figure S3. Quality controls for RNA sequencing and gene set enrichment analysis. Related to Figure 2. (A) FACS gating strategy for LN stromal cells. (B and C) Representative gels and histograms of (B) RNA and (C) cDNA samples from naïve and 4T1 tumor mice. (B) RNA quality as assessed by Agilent Bioanalyzer (RIN 7.8-10). (C) cDNA libraries with fragments between 300-500 bp length and without contaminations as tested on an Agilent TapeStation. (D) Relative expression of LEC-, BEC- and leukocyte-specific genes as measure for the high purity of sorted and sequenced LN LECs. (E) Gene set enrichment analysis of previously published sprouting tip cell-associated genes (del Toro et al., 2010; Strasser et al., 2010) among the differentially expressed genes in LN LECs in the 4T1 (left panel) and B16F10 (right panel) tumor models.

Figure S4:

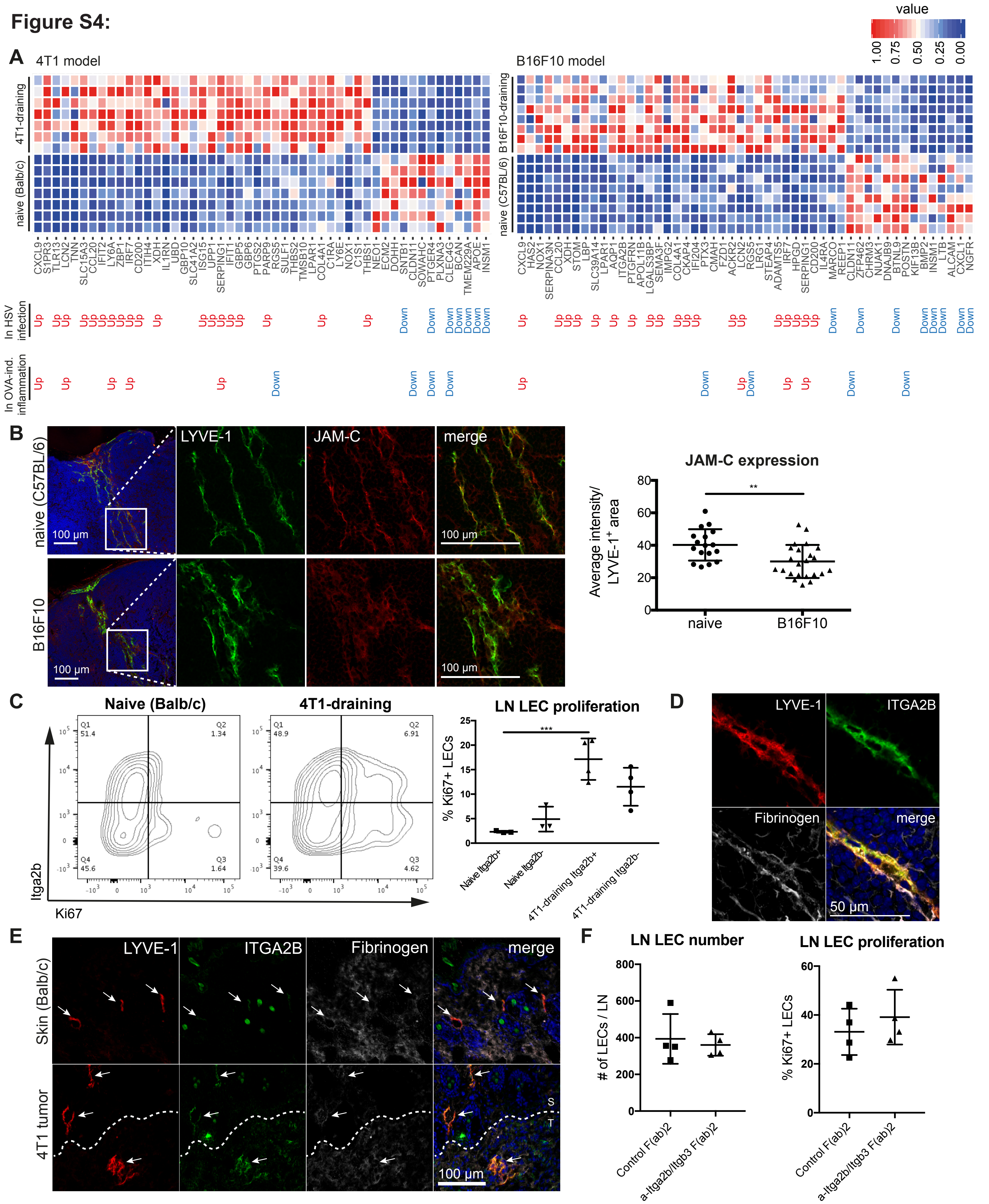
A
4T1 model
B16F10 model
value
1.00 0.75 0.50 0.25 0.00
4T1-draining
naive (Balb/c)
B16F10-draining
naive (C57BL/6)
In HSV infection
In OVA-ind. inflammation
B
LYVE-1
JAM-C
merge
naive (C57BL/6)
B16F10
100 µm
JAM-C expression
Average intensity/ LYVE-1+ area
naive
B16F10
C
Naive (Balb/c)
4T1-draining
Itga2b
Ki67
LN LEC proliferation
% Ki67+ LECs
Naive Itga2b+
Naive Itga2b-
4T1-draining Itga2b+
4T1-draining Itga2b-
D
LYVE-1
ITGA2B
Fibrinogen
merge
50 µm
E
LYVE-1
ITGA2B
Fibrinogen
merge
Skin (Balb/c)
4T1 tumor
100 µm
F
LN LEC number
# of LECs / LN
LN LEC proliferation
% Ki67+ LECs
Control F(ab)2
a-Itga2b/Itgb3 F(ab)2

Figure S4. Differential gene expression in LECs from tumor-draining LNs compared to naïve controls. Related to Figures 3 and 4.

(A) Heat map of top 50 most differentially expressed genes is shown for both tumor models separately (left: 4T1 vs. naïve Balb/c; right: B16F10 vs. naïve C57BL/6). Significant up- or downregulation in previously published gene expression datasets of LN LECs after HSV infection (Gregory et al., 2017) or after ovalbumin injection in OT-1 transferred mice (Malhotra et al., 2012) is indicated. (B) Representative immunofluorescence images and quantification of JAM-C staining intensity within LYVE-1 positive lymphatic structures of naïve and B16F10 tumor-draining LNs. Each dot represents one image, n = 8 individual LNs per group. Statistical significance was determined by the unpaired Student's t-test. (C) FACS analysis of LN LECs in naïve Balb/c and 4T1-bearing mice (day 10) for expression of integrin αIIb and Ki67. Example plots (left panels) and quantification (right panels, n = 3-4 mice / group) are shown. (D) Representative immunofluorescence image of fibrinogen localization around an integrin αIIb expressing lymphatic vessel in a 4T1 draining LN (day 20). (E) Representative immunofluorescence images of integrin αIIb expression and fibrinogen deposition in lymphatic vessels in control skin and primary 4T1 tumors (day 20). (F) FACS analysis of draining LN LEC numbers and proliferation rate (as determined by Ki67 staining) in 4T1-bearing mice (day 10) treated daily from day 4 on with a rat IgG2b F(ab)2 fragment or an integrin αIIb blocking F(ab)2 fragment.

Table S1:

		FACS sorted cell number				RNA extraction			DNA library prep	
	sample	LEC	BEC	FRC	TN	RIN	RNA conc. [pg/µl]	total RNA [ng]	DNA conc. [pg/µl]	Molarity [nM]
	N1	5016	8431	/	/	8.6	251	2.51	6350	24.40
	N2	2076	10135	/	/	9.9	234	2.34	5480	23.00
	N3	3105	8532	/	/	8.6	459	4.59	2630	10.60
Balb/c	N4	4225	10299	/	/	7.8	221	2.21	5130	20.00
	N5	2768	11378	10034	68533	8.7	499	4.99	820	3.15
	N6	3049	8810	12110	57358	9	660	6.60	2970	11.60
	N7	2788	8985	9794	66826	8.4	548	5.48	4450	17.20
	average	3290	9510	10646	64239					
	T1	1832	7569	/	/	9.8	382	3.82	806	3.31
	T2	2399	6682	/	/	9.3	462	4.62	1690	6.92
	T3	2201	6358	/	/	10	401	4.01	3320	13.40
4T1	T4	2487	5443	/	/	7.9	709	7.09	1960	7.50
	T5	1313	5536	5063	89048	8.8	236	2.36	4280	16.50
	T6	2293	5987	6139	91335	8	535	5.35	3310	13.40
	T7	2719	6064	7999	94794	9.1	384	3.84	4170	15.00
	average	2178	6234	6400	91726					
	N1	1415	7762	5866	11482	8.4	238	2.38	310	1.24
	N2	3193	10056	8735	65806	9.3	1033	10.33	2080	7.98
	N3	1925	10391	3299	21926	9.0	481	4.81	2660	10.70
C57BL/6	N4	1024	1856	929	14359	7.9	199	1.99	2080	8.71
	N5	2134	13047	3716	68532	8.4	357	3.57	3710	15.20
	N6	1967	12181	5175	50218	8.3	667	6.67	1080	4.42
	N7	2389	11294	4640	62914	8.5	727	7.27	5390	22.40
	N8	3006	7600	5142	42456	8.6	709	7.09	9	0.04
	average	2132	9273	4688	42212					
	T1	1524	5490	5363	19391	9.5	369	3.69	2350	9.48
	T2	719	3995	2991	15776	8.6	280	2.80	1900	7.52
	T3	2653	5131	11385	95043	9.5	1035	10.35	2200	9.14
B16F10	T4	3071	7339	6376	103462	9.5	1015	10.15	2920	11.60
	T5	1849	8883	4414	64414	8.9	592	5.92	3410	14.20
	T6	3228	12168	7462	32195	9.6	816	8.16	8900	35.10
	T7	1860	5835	3227	61444	8.9	547	5.47	3670	13.90
	T8	3045	11057	7245	117991	8.7	688	6.88	6370	26.00
	average	2244	7487	6058	63715					

Table S1. List of all samples used for RNA sequencing. Related to Figure 2.
Number of sorted LN stromal cells, LEC RNA quality (RIN) and quantity, and cDNA concentration in the libraries are shown for all samples used in this study. n = 7-8 samples per group.

Table S2:

Cluster 1

BP (Biological Process)

Term	ID	p	N
immune system process	GO:0002376	7.252306e-09	18/187
positive regulation of macrophage activation	GO:0043032	3.531137e-06	4/7
innate immune response	GO:0045087	3.699133e-07	15/168
cellular response to interferon-gamma	GO:0071346	1.036803e-06	6/20
cell adhesion	GO:0007155	5.197905e-07	24/247
defense response	GO:0006952	9.695147e-06	5/17
adhesion of symbiont to host	GO:0044406	1.235389e-05	4/9
negative regulation of endothelial cell proliferation	GO:0001937	3.005367e-05	5/21
negative regulation of angiogenesis	GO:0016525	3.461842e-05	6/35

Molecular Function (MF)

Term	ID	p	N
extracellular matrix binding	GO:0050840	5.97E-06	5/16
scavenger receptor activity	GO:0005044	1.52E-05	5/19
calcium ion binding	GO:0005509	2.55E-05	15/244
chemorepellent activity	GO:0045499	5.23E-05	3/5
collagen binding	GO:0005518	5.51E-05	6/39
extracellular matrix structural constituent	GO:0005201	8.10E-05	4/14

Cellular Composition (CC)

Term	ID	p	N
extracellular region	GO:0005576	1.39104E-11	33/471
cell surface	GO:0009986	6.5854E-11	25/289
membrane	GO:0016020	5.0668E-09	109/3895
plasma membrane	GO:0005886	2.13435E-07	58/1664
extracellular matrix	GO:0031012	1.13981E-05	10/99
proteinaceous extracellular matrix	GO:0005578	1.81583E-05	11/127
endoplasmic reticulum	GO:0005783	5.45915E-05	32/853

Cluster 2

BP (Biological Process)

Term	ID	p	N
response to cAMP	GO:0051591	6.61424E-06	5/14
SMAD protein signal transduction	GO:0060395	3.74705E-05	6/31
positive regulation of cell differentiation	GO:0045597	4.62223E-05	5/20
transcription, DNA-templated	GO:0006351	6.83504E-05	46/1242
transcription from RNA polymerase II promoter	GO:0006366	1.7366E-05	17/248
negative regulation of transcription	GO:0000122	1.18991E-08	31/471
positive regulation of transcription	GO:0045944	2.49249E-08	35/598
regulation of transcription, DNA templated	GO:0006355	5.73019E-06	51/1308
response to mechanical stimulus	GO:0009612	8.13038E-05	4/12

Molecular Function (MF)

Term	ID	p	N
transcription factor activity, sequence-specific DNA binding	GO:0003700	5.08779E-07	27/436
transcriptional activator activity	GO:0001077	7.30895E-05	11/126
protein binding	GO:0005515	2.70703E-05	88/2845
DNA binding	GO:0003677	5.72488E-05	41/1036
sequence-specific DNA binding	GO:0043565	1.19057E-06	20/271

Cellular Composition (CC)

Term	ID	p	N
nuclear chromatin	GO:0000790	6.73611E-05	11/127

Cluster 3

BP (Biological Process)

Term	ID	p	N
response to virus	GO:0009615	4.43297E-06	5/43
defense response to virus	GO:0051607	7.22143E-10	9/81
cellular response to interferon-beta	GO:0035458	5.61888E-06	4/21
G-protein coupled receptor signaling pathway	GO:0007186	1.00859E-05	7/132
immune system process	GO:0002376	1.0928E-05	8/187
inflammatory response	GO:0006954	5.49557E-05	6/117

Molecular Function (MF)

Term	ID	p	N
chemokine activity	GO:0008009	5.44971E-05	3/13

Cellular Composition (CC)

Term	ID	p	N
extracellular region	GO:0005576	2.33269E-06	13/471

Cluster 4

BP (Biological Process): N/A

Molecular Function (MF): N/A

Cellular Composition (CC): N/A

Cluster 5

BP (Biological Process): N/A

Molecular Function (MF): N/A

Cellular Composition (CC)

Term	ID	p	N
extracellular space	GO:0005615	1.93289E-05	6/462

Cluster 6

BP (Biological Process)

Term	ID	p	N
cell division	GO:0051301	1.20509E-05	5/259
mitotic nuclear division	GO:0007067	8.49154E-05	4/194

Molecular Function (MF): N/A

Cellular Composition (CC): N/A

Table S2. Complete list of gene ontologies (GOs) associated with the clusters in Figure 3A. Related to Figure 3.

Movie S1. Optical sectioning reveals the lymphatic network architecture in resting LNs of naïve mice. Related to Figure 1.
Consecutive 3D light-sheet microscope images of a whole naïve LN stained for the lymphatic marker LYVE-1.

Movie S2. Optical sectioning reveals altered lymphatic network architecture in 4T1 tumor-draining LNs. Related to Figure 1.
Consecutive 3D light-sheet microscope images of a whole 4T1 tumor-draining LN stained for the lymphatic marker LYVE-1.
